# Efficacy and breadth of adjuvanted SARS-CoV-2 receptor-binding domain nanoparticle vaccine in macaques

**DOI:** 10.1073/pnas.2106433118

**Published:** 2021-09-01

**Authors:** Hannah A. D. King, M. Gordon Joyce, Ines Lakhal-Naouar, Aslaa Ahmed, Camila Macedo Cincotta, Caroline Subra, Kristina K. Peachman, Holly R. Hack, Rita E. Chen, Paul V. Thomas, Wei-Hung Chen, Rajeshwer S. Sankhala, Agnes Hajduczki, Elizabeth J. Martinez, Caroline E. Peterson, William C. Chang, Misook Choe, Clayton Smith, Jarrett A. Headley, Hanne A. Elyard, Anthony Cook, Alexander Anderson, Kathryn McGuckin Wuertz, Ming Dong, Isabella Swafford, James B. Case, Jeffrey R. Currier, Kerri G. Lal, Mihret F. Amare, Vincent Dussupt, Sebastian Molnar, Sharon P. Daye, Xiankun Zeng, Erica K. Barkei, Kendra Alfson, Hilary M. Staples, Ricardo Carrion, Shelly J. Krebs, Dominic Paquin-Proulx, Nicos Karasavvas, Victoria R. Polonis, Linda L. Jagodzinski, Sandhya Vasan, Paul T. Scott, Yaoxing Huang, Manoj S. Nair, David D. Ho, Natalia de Val, Michael S. Diamond, Mark G. Lewis, Mangala Rao, Gary R. Matyas, Gregory D. Gromowski, Sheila A. Peel, Nelson L. Michael, Kayvon Modjarrad, Diane L. Bolton

**Affiliations:** ^a^US Military HIV Research Program, Walter Reed Army Institute of Research, Silver Spring, MD 20910;; ^b^Emerging Infectious Diseases Branch, Walter Reed Army Institute of Research, Silver Spring, MD 20910;; ^c^Henry M. Jackson Foundation for the Advancement of Military Medicine, Bethesda, MD 20817;; ^d^Diagnostics and Countermeasures Branch, Walter Reed Army Institute of Research, Silver Spring, MD 20910;; ^e^Viral Diseases Branch, Walter Reed Army Institute of Research, Silver Spring, MD 20910;; ^f^Department of Medicine, Washington University, St. Louis, MO 63130;; ^g^Department of Pathology & Immunology, Washington University, St. Louis, MO 63130;; ^h^Center for Molecular Microscopy, Center for Cancer Research, National Cancer Institute, NIH, Frederick, MD 21702;; ^i^BIOQUAL, Inc., Rockville, MD 20850;; ^j^Center for Infectious Diseases Research, Walter Reed Army Institute of Research, Silver Spring, MD 20910;; ^k^Division of Pathology, US Army Medical Research Institute of Infectious Diseases, Frederick, MD 21702;; ^l^Veterinary Pathology Branch, Walter Reed Army Institute of Research, Silver Spring, MD 20910;; ^m^Disease Intervention and Prevention, Texas Biomedical Research Institute, San Antonio, TX 78227;; ^n^Aaron Diamond AIDS Research Center, Columbia University Vagelos College of Physicians and Surgeons, New York, NY 10032;; ^o^Department of Molecular Microbiology, Washington University, St. Louis, MO 63130

**Keywords:** SARS-CoV-2, vaccine, macaque, nanoparticle, adjuvant

## Abstract

Emergence of novel variants of the severe acute respiratory syndrome coronavirus 2 (SARS-CoV-2) underscores the need for next-generation vaccines able to elicit broad and durable immunity. Here we report the evaluation of a ferritin nanoparticle vaccine displaying the receptor-binding domain of the SARS-CoV-2 spike protein (RFN) adjuvanted with Army Liposomal Formulation QS-21 (ALFQ). RFN vaccination of macaques using a two-dose regimen resulted in robust, predominantly Th1 CD4+ T cell responses and reciprocal peak mean serum neutralizing antibody titers of 14,000 to 21,000. Rapid control of viral replication was achieved in the upper and lower airways of animals after high-dose SARS-CoV-2 respiratory challenge, with undetectable replication within 4 d in seven of eight animals receiving 50 µg of RFN. Cross-neutralization activity against SARS-CoV-2 variant B.1.351 decreased only approximately twofold relative to WA1/2020. In addition, neutralizing, effector antibody and cellular responses targeted the heterotypic SARS-CoV-1, highlighting the broad immunogenicity of RFN-ALFQ for SARS-CoV−like Sarbecovirus vaccine development.

## Significance

The emergence of SARS-CoV-2 variants of concern (VOCs) that reduce the efficacy of current COVID-19 vaccines is a major threat to pandemic control. We evaluate a SARS-CoV-2 spike receptor-binding domain ferritin nanoparticle protein vaccine (RFN) in a nonhuman primate challenge model that addresses the need for a next-generation vaccine with increased pan-SARS breadth of coverage. RFN, adjuvanted with a liposomal-QS21 formulation (ALFQ), elicits humoral and cellular immune responses with excellent breadth and potency against SARS-CoV-2 VOCs and SARS-CoV-1, and protects against high-dose respiratory tract challenge with SARS-CoV-2. Our results support consideration of RFN for vaccine development against multiple concerning members of the Sarbecovirus subgenus of *Betacoronaviruses*.

The COVID-19 pandemic, precipitated by severe acute respiratory syndrome coronavirus 2 (SARS-CoV-2), continues to threaten global public health and economies. Threats of future outbreaks also loom, as evidenced by three emergent SARS-like diseases caused by zoonotic *Betacoronaviruses* in the last two decades. While several emergency use authorized (EUA) vaccines currently in use are expected to curb both disease and transmission of SARS-CoV-2 (1–6), the emergence of circulating variants of concern (VOCs) less sensitive to vaccine-elicited immunity has raised concerns for sustained vaccine efficacy (7). Logistical challenges of vaccine production, distribution, storage, and access for these vaccines must be resolved to achieve resolution to the pandemic (8, 9). The rapid and unparalleled spread of SARS-CoV-2 has driven an urgent need to deploy scalable vaccine platforms to combat the ongoing pandemic and mitigate future outbreaks.

Current vaccines primarily focus the immune response on the spike glycoprotein (S) as it mediates host cell viral fusion and entry. The receptor-binding domain (RBD) of S engages the primary host cell receptor, angiotensin-converting enzyme 2 (ACE2), for both SARS-CoV-2 and SARS-CoV-1, making RBD a promising domain for vaccine-elicited immune focus (10–12). Moreover, many of the potently neutralizing monoclonal antibodies isolated against SARS-CoV-2 target the RBD (13, 14). Vaccination of nonhuman primates (NHPs) with RBD-encoding RNA or DNA protects against respiratory tract challenge, indicating that immune responses to the RBD can prevent viral replication (15, 16). RBD vaccination also elicits cross-reactive responses to circulating SARS-CoV-2 VOCs in both animals and humans (17, 18), with decrements against the B.1.351 variant similar to that seen with S immunogens (19). The breadth of RBD immunogenicity is further supported by the ability of RBD-specific monoclonal antibodies isolated from SARS-CoV-1 convalescent individuals to cross-neutralize SARS-CoV-2 (20, 21). These findings suggest potential for RBD-based vaccines being efficacious against SARS-CoV-2 variants and other related coronavirus species.

Approaches to improve immunogenicity of S or RBD protein vaccines include optimizing antigen presentation and coformulating with adjuvants to enhance the protective immunity. One common approach to enhance the induction of adaptive immune responses is the multimeric presentation of antigen, for example, on the surface of nanoparticles or virus-like particles (22). Presenting RBD in ordered, multivalent arrays on the surface of self-assembling protein nanoparticles is immunogenic and efficacious in animals (23–28), with improved immunogenicity relative to monomeric soluble RBD and cross-reactive responses to variants (17, 24, 26). However, it is unknown whether RBD nanoparticle vaccines protect against infection in primates, which have become a standard model for benchmarking performance of vaccine candidates by virological and immunologic endpoints. Liposomal adjuvants incorporating QS-21, such as that used in the efficacious varicella zoster vaccine, SHINGRIX, may augment protective immunity to SARS-CoV-2 vaccines. Such adjuvants have superior humoral and cellular immunogenicity relative to conventional adjuvants (29, 30).

Here, we evaluate the use of a ferritin nanoparticle vaccine presenting the SARS-CoV-2 RBD (RFN) adjuvanted with the Army Liposomal Formulation QS-21 (ALFQ) (31). Both ferritin nanoparticles and ALFQ have been evaluated for vaccination against multiple pathogens in humans in phase 1 clinical trials (32–34). We demonstrate, in an NHP model, that immunization with RFN induces robust and broad antibody and T cell responses, as well as protection against viral replication and lung pathology following high-dose respiratory tract challenge with SARS-CoV-2.

## Results

### Vaccine and Animal Study Design.

**Fig. 1.**
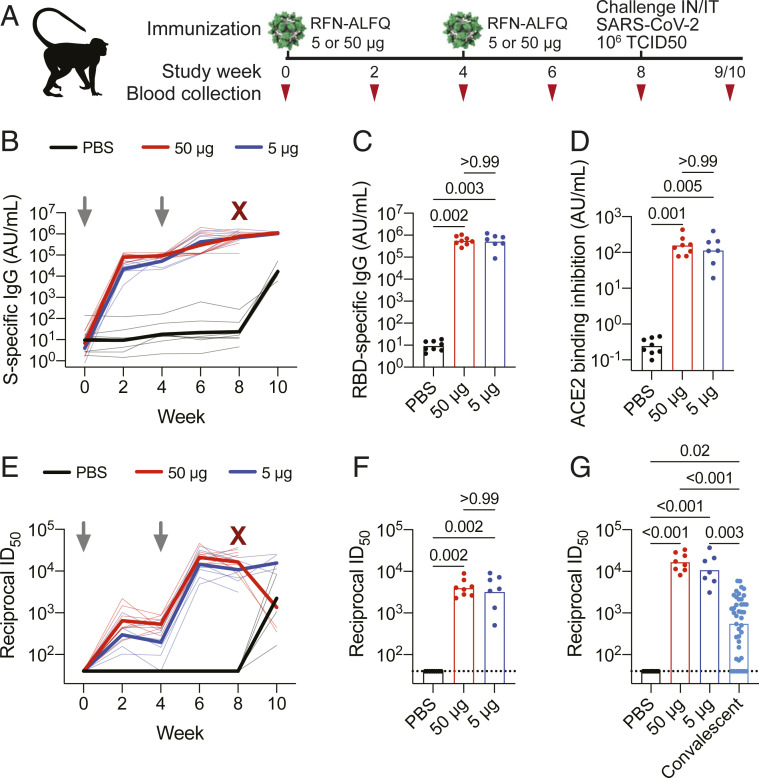
RFN vaccine−elicited binding and neutralizing antibody responses to SARS-CoV-2. Humoral immune responses were measured in vaccinated macaques. (*A*) Rhesus macaque vaccination, challenge, and sampling schedule. Animals were immunized with either 50 μg or 5 µg of RFN-ALFQ at weeks 0 and 4; control animals received PBS (*N* = 7 or 8 per group); 1 × 10^6^ TCID_50_ of SARS-CoV-2 was administered 4 wk after the last vaccination. (*B*) Serum SARS-CoV-2 S-specific IgG responses assessed by MSD immunoassay every 2 wk following vaccination. Data are depicted as arbitrary units per milliliter of IgG binding. Thick lines indicate geometric means within each group, and thin lines represent individual animals. Serum SARS-CoV-2 RBD-specific IgG (*C*) and inhibition of ACE2 binding to the RBD (*D*) 4 wk after last vaccination were measured by MSD immunoassay. (*E*) Serum SARS-CoV-2 S-specific pseudovirus neutralization every 2 wk following vaccination. Virus neutralization reciprocal ID_50_ is shown. Thick lines indicate geometric means within each group, and thin lines represent individual animals. (*F*) Authentic SARS-CoV-2 WA1/2020 virus neutralization at 4 wk after last vaccination. (*G*) Pseudovirus neutralization activity 4 wk postboost was compared to a panel of human convalescent sera (*n* = 41 samples). Bars indicate the geometric mean titer. Symbols represent individual animals and overlap with one another for equal values where constrained. In *B* and *E*, gray arrows indicate the time of immunization; maroon Xs indicate time of challenge. Significance was assessed using a Kruskal−Wallis test followed by a Dunn’s posttest.

A SARS-CoV-2 RBD ferritin nanoparticle vaccine (RFN) was designed as a ferritin-fusion recombinant protein that self-assembles into a 24-mer nanoparticle displaying a multivalent, ordered array of RBD on its surface. Briefly, the RBD protein sequence (residues 331 to 527) derived from the Wuhan-Hu-1 genome sequence (GenBank accession number MN908947.3) was covalently linked to the C-terminal region of the *Helicobacter pylori* ferritin molecule. Twenty-three rhesus macaques were immunized with either 50 μg or 5 μg of RFN, or sham-immunized with phosphate-buffered saline (PBS) (*n* = 7/8 per group), at study weeks 0 and 4 (Fig. 1*A*). RFN was adjuvanted with ALFQ, which contains synthetic monophosphoryl 3-deacyl lipid A and QS-21. Animals were challenged 4 wk after the last immunization via combined intratracheal (IT, 1.0 mL) and intranasal (IN, 0.5 mL per nostril) inoculation of a 10^6^ TCID_50_ (50% tissue culture infectious dose) dose of SARS-CoV-2 virus (WA1/2020). Animals were followed for 7 d (*n* = 12) or 14 d (*n* = 11) following challenge for immunological, virological, and pathologic assessments.

### Humoral Responses to Vaccination.

Multiple vaccine-matched humoral immune responses were measured longitudinally in serum following vaccination. First, binding antibody responses to the SARS-CoV-2 prefusion stabilized S protein (S-2P) (35) were assessed by Meso Scale Discovery (MSD) sandwich electrochemiluminescent immunoassay. Immunization with either 5 μg or 50 μg of RFN elicited S-specific IgG 2 wk following the prime (21,896 and 79,109 arbitrary units (AU)/mL, respectively) (Fig. 1*B*). These responses increased 2 wk following the second immunization (420,249 and 293,509 AU/mL). Boosting was greater with the 5-μg dose, achieving a 19-fold increase relative to postprime versus ∼3.7-fold with 50 μg. Responses continued to marginally increase 4 wk following the second immunization. Unvaccinated control animals mounted responses ∼1,000-fold over baseline within 2 wk postchallenge, and these responses were ∼65-fold lower than those in vaccinated animals after challenge.

Given the importance of the RBD in mediating viral entry and the majority of neutralizing antibody responses targeting this domain, RBD-specific humoral responses were also measured by MSD immunoassay. RFN induced binding antibodies 4 wk following the second immunization, with no significant difference between vaccine dose groups (Fig. 1*C*). Vaccinated animals developed RBD-specific IgG 4 wk following the prime (506,123 and 553,705 AU/mL in the 5- and 50-μg groups, respectively) comparable in magnitude to those against the S protein, consistent with an RBD-focused response. To confirm these findings, the on-rate association between serum antibodies and RBD antigen was measured by biolayer interferometry and longitudinal responses exhibited a similar profile as S-specific binding (*SI Appendix*, Fig. S1). Again, vaccine dose groups did not differ. Functional activity of serum antibodies to inhibit ACE2 binding to the RBD antigen was also measured and showed comparably high-magnitude responses elicited by RFN at both the 5- and 50-μg doses (Fig. 1*D*).

Neutralizing antibody responses against SARS-CoV-2 using a pseudovirus assay followed a pattern similar to the S-specific binding responses (Fig. 1*E*). Peak ID_50_ values (50% inhibitory dilution—the reciprocal of the serum dilution necessary to achieve 50% neutralization) of 14,540 and 21,298 were observed 2 wk following the boost for the 5- and 50-μg RFN doses, respectively. Neutralizing responses increased markedly between the prime and boost, rising 48- and 32-fold between study weeks 2 and 6. Among the 50-μg RFN−vaccinated animals followed 2 wk postchallenge, neutralizing responses declined 6 wk postboost by ∼1 log relative to peak values, indicating neutralizing responses may decay more quickly than binding antibodies.

Neutralizing responses were also evaluated using an authentic intact SARS-CoV-2 virus (WA1/2020 isolate) and a focus reduction neutralization assay (36). Robust neutralizing titers were detected in all RFN-vaccinated animals (Fig. 1*F*). Median ID_50_ values were ∼3,800 for both dose groups, though slightly more variable with 5-μg dosing. This result paralleled responses assessed by a pseudovirus assay (Fig. 1 *E* and *G*). Since serum from convalescent COVID-19 human cases is frequently used as a benchmarking reference for vaccine immunogenicity in clinical and preclinical studies, we compared RFN-vaccinated macaque pseudovirus neutralizing titers to those of 41 convalescent individuals 4 wk to 8 wk post−COVID-19. Responses in the 50-μg group were, on average, 13-fold higher than those of convalescent individuals, whereas titers from the 5-μg group were ∼10-fold higher, indicating that RFN-elicited neutralizing antibody activity exceeds that observed in the first months following human infection. Thus, RFN vaccination generated strong RBD-specific binding antibodies with potent neutralizing activity that blocks the interaction between the RBD and the host ACE2 receptor.

Nonneutralizing antibody effector functions may be associated with vaccine-mediated protection against viruses including SARS-CoV-2 (37–39). Strong IgG-mediated cellular opsonization responses were observed following the second immunization, whereas IgM and IgA responses were more modest (*SI Appendix*, Fig. S2 *A*–*C*). Serum antibody-dependent phagocytosis mediated by either monocytes (antibody-dependent cellular phagocytosis **[**ADCP]) or neutrophils (antibody-dependent neutrophil phagocytosis **[**ADNP]) as well as complement deposition (antibody-dependent complement deposition **[**ADCD]) responses were also robust in both vaccinated groups and consistently peaked at week 6 (*SI Appendix*, Fig. S2 *D*–*F*). A similar pattern was seen for antibody-dependent trogocytosis (40) (*SI Appendix*, Fig. S2*G*). Overall, 5 µg of RFN achieved equal Fc-mediated effector functions compared to 50 µg, although ADCD responses trended ∼1.25-fold greater with the higher dose.

### Virus-Specific T Cell Responses.

**Fig. 2.**
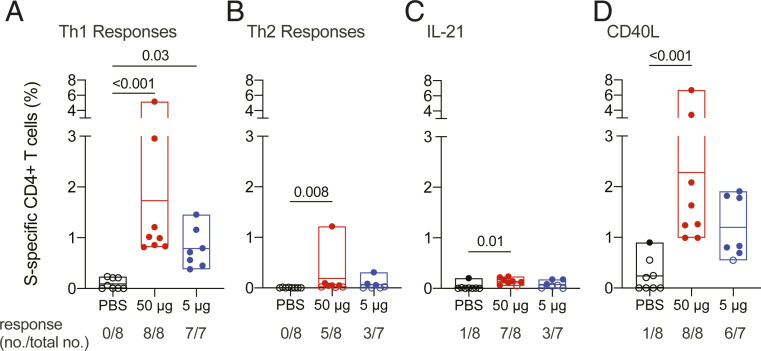
RFN vaccine−elicited SARS-CoV-2 S-specific CD4+ T cell responses. T cell responses were assessed by SARS-CoV-2 S peptide pool stimulation and intracellular cytokine staining of PBMCs collected 4 wk after last vaccination. S-specific memory CD4+ T cells expressing the indicated markers are shown as follows: (*A*) Th1 cytokines (IFNγ, TNF, and IL-2), (*B*) Th2 cytokines (IL-4 and IL-13), (*C*) IL-21, and (*D*) CD40L. Boolean combinations of cytokine-positive memory CD4+ T cells were summed in *A* and *B*. Probable positive responses, defined as >3 times the group background at baseline, are depicted as closed symbols. Positivity rates within each group are shown below each graph as a fraction. Box plot horizontal lines indicate the mean; top and bottom reflect the minimum and maximum. Significance was assessed using a Kruskal−Wallis test followed by a Dunn’s posttest.

SARS-CoV-2−specific T cell immunity is associated with reduced disease severity and can influence antibody responses (41, 42). We assessed S-specific T cells in peripheral blood mononuclear cells (PBMCs) by in vitro peptide stimulation and intracellular cytokine staining using a 19-color multiparameter flow cytometry panel for detailed functional characterization of T cell responses from RFN vaccination. A vigorous, dose-dependent Th1 (TNF, IL-2, IFN-γ) CD4+ T cell response was observed in all RFN-vaccinated animals 4 wk after the second vaccination, ranging from 0.4 to 5.2% of memory cells (Fig. 2*A*). These S-specific Th1 cells were polyfunctional in quality, a property associated with control of other pathogens (43), as a large proportion concurrently expressed multiple Th1 cytokines (*SI Appendix*, Fig. S3*A*). Th2 responses were low or undetectable (Fig. 2*B*), with median Th1/Th2 ratios of ∼20 among 50-µg−vaccinated animals with evidence of a Th2 response (*SI Appendix*, Fig. S3*B*). Modest CD8+ T cell responses were observed in about half of the animals and were more prominent in recipients of 50 μg than 5 μg of RFN (*SI Appendix*, Fig. S3*C*). Response magnitude was ∼0.1 to 0.4% of memory CD8+ T cells.

Additional CD4+ T cell functions important for the development of antibody responses were evaluated. S-specific CD4+ T cell IL-21 responses, a surrogate marker of peripheral T follicular helper cell activity, were observed in the majority of animals vaccinated with 50 µg of RFN and in half of the animals vaccinated with 5 µg of RFN (Fig. 2*C*). The average frequency in responders was 0.15%. The CD4+ T cell activation marker, CD40L, which promotes B cell antibody isotype switching, was highly expressed by S-specific cells (Fig. 2*D*). Responses ranged from ∼1 to 7% after 50 μg of RFN and were observed in all eight animals, whereas response rates and magnitude were slightly reduced with the 5-µg dose (∼0.7 to 2% in six of seven animals). Overall, these data show that adjuvanted RFN induced robust Th1-polarized polyfunctional CD4+ T cells favorable for viral clearance and with critical B cell help capability.

### SARS-CoV-2 Challenge Efficacy and Immune Correlates of Protection.

**Fig. 3.**
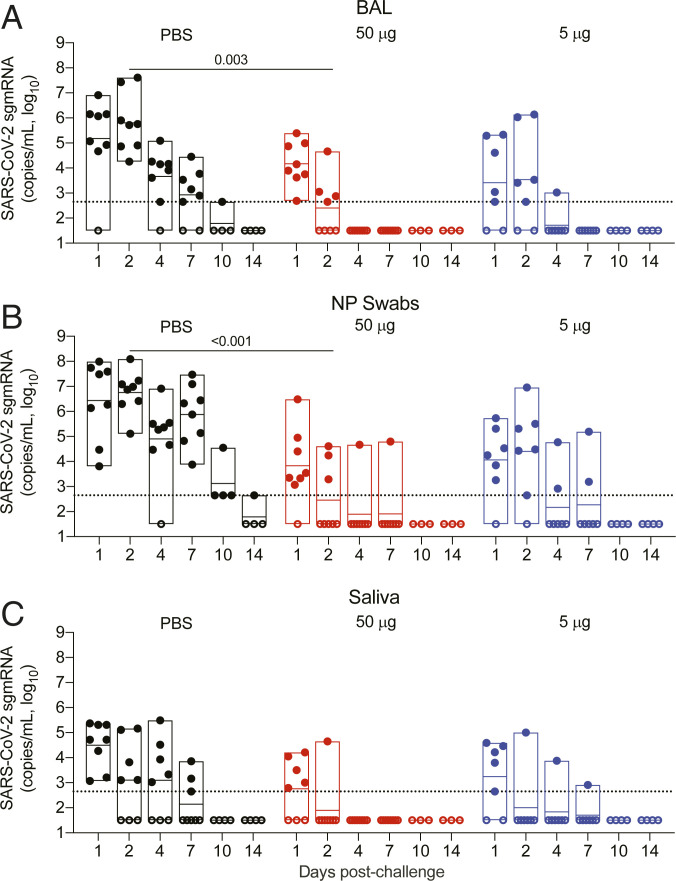
Viral replication in the lower and upper airways after RFN vaccination and subsequent SARS-CoV-2 respiratory challenge. The sgmRNA for the E (Envelope) target (copies per milliliter) were measured following challenge in (*A*) BAL, (*B*) NP swabs, and (*C*) saliva of vaccinated and control animals for 1 wk (*n* = 7 or 8 per group) or 2 wk (*n* = 3 or 4 per group) following IN and IT SARS-CoV-2 (WA1/2020) challenge of vaccinated and control animals. Specimens were collected 1, 2, 4, 7, 10, and 14 d postchallenge. Dotted lines demarcate assay lower limit of linear performance range (log 10 of 2.65 corresponding to 450 copies per mL); positive values below this limit are plotted as 450 copies per mL. Open symbols represent animals with viral loads below the limit of detection of the assay. Box plot horizontal lines indicate the mean; top and bottom reflect the minimum and maximum. Significant differences between control and vaccinated animals at day 2 postchallenge are indicated. Significance was assessed using a Kruskal−Wallis test followed by a Dunn’s posttest.

To assess the protective efficacy of RFN vaccination, animals were challenged with high-dose (10^6^ TCID_50_) SARS-CoV-2 WA1/2020 administered via the simultaneous IN/IT routes 4 wk following the second immunization. The presence of viral RNA was assessed in both the upper (nasopharyngeal [NP] swabs and saliva) and lower (bronchoalveolar lavage fluid [BAL]) respiratory tract. Measurements were made of both total RNA and subgenomic E messenger RNA (sgmRNA), the latter considered a more specific indicator of viral replication (44, 45). Unvaccinated control animals all showed evidence of a robust infection, with mean levels of sgmRNA in the BAL of ∼10^6^ copies per mL, and, in the NP swabs, of ∼10^7^ copies per mL at day 2 postchallenge (Fig. 3). Moreover, viral replication was sustained at >10^4^ copies sgmRNA per mL for 7 d in the upper airways. In RFN-vaccinated animals, the magnitude and duration of viral replication was markedly reduced. In the 50-μg group, day 1 sgmRNA was reduced by 1 and 2 logs in the BAL and NP swabs, respectively. Rapid clearance was observed by day 2 in five of eight animals in the upper airways and four of eight in the lower airways. Both airways were void of replicating virus in all but one animal by day 4. Viral control was also apparent after 5-μg RFN vaccination. Reductions in BAL and NP swab sgmRNA on day 1 were similar to that achieved with 50 μg, while more breakthrough replication was apparent 2 d after challenge. Clearance was observed by day 4 in five of seven animals in the upper airways and six of seven in the lower airways. Replicating virus was absent in the airways in all but two animals by day 7.

Viral replication was detected in saliva in all control animals on day 1 and persisted in five animals through day 4 (Fig. 3*C*). Values were lower than those in BAL or NP swabs and tapered to undetectable levels more rapidly. Fewer vaccinated animals contained sgmRNA in their saliva, and replicating virus was detected in only one animal from each vaccine dose group starting on day 2. The kinetics of SARS-CoV-2 total RNA, which is more likely to reflect material from the viral inoculum, paralleled results described above for sgmRNA in BAL, NP swabs, and saliva (*SI Appendix*, Fig. S4).

Development of adaptive immune responses against SARS-CoV-2 following challenge can also provide evidence of viral replication in infected animals. Antibodies to the viral nucleocapsid (N) protein, which is not present in the vaccine, were measured at study weeks 9 and 10 using the MSD immunoassay. Unvaccinated control animals developed N-specific binding IgG responses within 2 wk following challenge, while responses were not observed in RFN-vaccinated animals (*SI Appendix*, Fig. S5). This suggests absence of a de novo response to the challenge virus in the vaccine groups and is consistent with limited viral replication.

To identify vaccine-elicited immune responses that contribute to control of viral replication following challenge, we performed an exploratory immune correlates analysis. SARS-CoV-2−specific humoral and cellular immune responses among RFN-vaccinated animals were assessed for inverse associations with viral replication in the upper respiratory tract, as represented by viral sgmRNA in NP swabs 2 d postchallenge, when the greatest variation in viral burden was observed. Several humoral responses at the time of challenge were inversely associated with viral clearance: IgG opsonization (rho = −0.63, *P* = 0.015), IgA opsonization (rho = −0.53, *P* = 0.046), and ADCD (rho = −0.56, *P* = 0.032; Spearman correlation; two-tailed test). Trends were also observed for serum inhibition of ACE2 binding to S (rho = −0.52, *P* = 0.05) and the RBD (rho = −0.46, *P* = 0.088), as well as pseudovirus neutralization titers (rho = −0.46, *P* = 0.089) (*SI Appendix*, Fig. S6 *A*–*F*). Neither S-specific CD4+ or CD8+ T cell responses (*SI Appendix*, Fig. S6 *G*–*I*) nor authentic intact virus neutralization were associated with rapid clearance. These data indicate that serum humoral responses, including those with effector functions, may serve as a useful immune correlate of protection.

### Respiratory Tract Pathology and Antigen Expression.

**Fig. 4.**
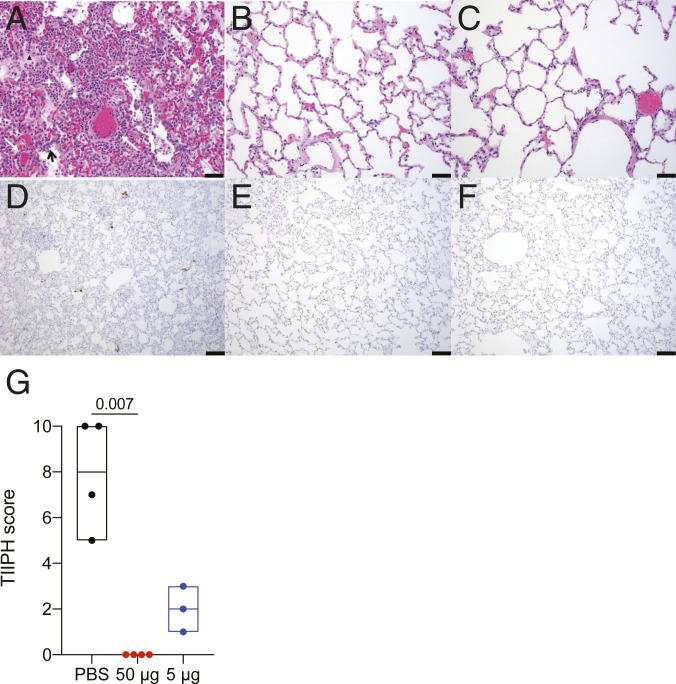
Histopathology and virus detection in the lungs following SARS-CoV-2 challenge. Lung parenchymal tissue was assessed for pathology and viral antigen 7 d postchallenge. (*A–C*) Paraffin-embedded sections were stained with hematoxylin and eosin and shown for animals that received PBS (*A*), 50 µg of RFN (*B*) and 5 μg of RFN (*C*). Inflammatory debris (white star), type II pneumocyte hyperplasia (black arrow), edema (black triangle), and vasculitis of small- to medium-caliber blood vessels (white arrows) is indicated. (Scale bars, 50 µm.) (*D*–*F*) SARS-CoV-2 nucleocapsid detected by IHC in alveolar pneumocytes, pulmonary macrophages, and endothelial cells appears as brown aggregates. (Scale bars, 100 µm.) Representative images are shown. (*G*) Each pathologic finding was quantified for six lung sections and reported as a combined TIIPH score for all animals necropsied 7 d postchallenge.

Vaccine efficacy was also assessed by histopathological analysis of lung tissue from three to five macaques from each group necropsied at day 7 postchallenge. By this point, all unvaccinated animals had developed evidence of multifocal, mild to moderate interstitial pneumonia (Fig. 4*A*). The pneumonia was characterized by type II pneumocyte hyperplasia, alveolar edema, alveolar inflammatory and necrotic debris, thickening of alveolar septae, increased numbers of pulmonary macrophages (including multinucleated giant cells), and vasculitis of small- to medium-caliber blood vessels. The middle and caudal lung lobes were most severely affected in all four unvaccinated animals. Histological evidence of interstitial pneumonia was not observed in animals from any of the vaccinated groups (Fig. 4 *B* and *C*). However, in each of the vaccine groups, there was minimal to mild mononuclear to mixed cellular infiltrates centered on small- to medium-caliber blood vessels. Immunohistochemistry (IHC) demonstrated SARS-CoV-2 viral antigen in small numbers of alveolar pneumocytes and macrophages in at least one lung section of every unvaccinated animal (Fig. 4*D*). No viral antigen was detected in the lungs of any of the animals in any of the vaccine groups (Fig. 4 *E* and *F*). Overall, pathological findings were significantly reduced by vaccination (Fig. 4*G*). No significant histopathologic differences were observed between vaccinated and unvaccinated animals at day 14, consistent with transient SARS-CoV-2 pathology in this model. Mild perivascular infiltrates occasionally remained in some animals from all groups. In summary, vaccination with 5 μg or 50 μg of RFN prevented moderate disease and viral protein expression in the lungs.

### Cross-Reactive Immunity to Emergent SARS-CoV-2 Variants and SARS-CoV-1.

**Fig. 5.**
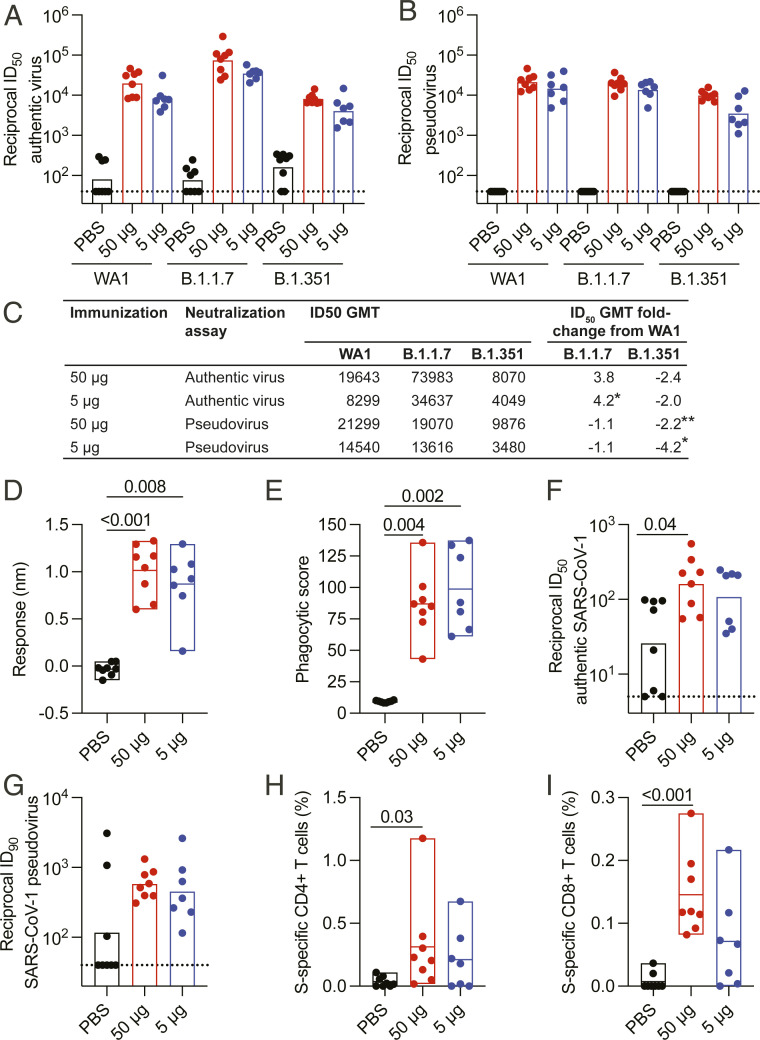
Cross-reactive immune responses to SARS-CoV-2 variants and SARS-CoV-1. Serum and PBMC collected 2 wk after the last vaccination was assessed for cross-reactivity to VOCs and SARS-CoV-1. (*A*) Authentic virus and (*B*) pseudovirus neutralizing antibody responses to variants B.1.1.7 and B.1.351. Corresponding responses to SARS-CoV-2 WA1/2020 authentic virus and Wuhan-1 pseudovirus are shown. Bars indicate the GMT. (*C*) Reciprocal ID_50_ GMT fold change from wild-type neutralization (WA1 or Wuhan-1) with statistical significance indicated (*P* < 0.05, *; *P* < 0.01, **). (*D*) Serum binding responses to SARS-CoV-1 RBD assessed by biolayer interferometry. (*E*) Antibody-dependent cellular phagocytosis of SARS-CoV-1 S trimer-coated fluorescent beads. (*F*) Authentic SARS-CoV-1 (Urbani) neutralization titers (ID_50_). (*G*) SARS-CoV-1 (Urbani) pseudovirus neutralization (ID_90_). (*H*) SARS-CoV-1 (Urbani) S-specific memory CD4+ Th1 and (*I*) CD8+ responses assessed by peptide pool stimulation and ICS (INFγ, IL-2, and TNF). Significance was assessed with a Kruskal−Wallis test followed by a Dunn’s posttest. Bars indicate the GMT.

Given concerns about increased resistance of circulating SARS-CoV-2 viral variants to currently available vaccines, we assessed serum from RFN-vaccinated macaques for neutralizing antibody responses against two VOCs, B.1.1.7 and B.1.351. In an authentic virus neutralization assay (46), reciprocal neutralization ID_50_ geometric mean titer (GMT) against B.1.1.7 were 73,983 2 wk following the second 50-μg dose (Fig. 5*A*). This translated to ∼3.8-fold greater titers than those against the wild-type, vaccine-matched WA1/2020 strain. Activity against the two strains was similar when measured by the pseudovirus neutralization assay (Fig. 5*B*). B.1.1.7 cross-reactive responses were observed regardless of vaccine dose, although titers trended lower with 5 µg of RFN. Neutralizing GMTs against B.1.351 decreased approximately twofold to 8,070 and 9,876 in the 50-μg-dose group in the authentic virus and pseudovirus assays, respectively, indicating only a minor diminution in potency compared to WA1/2020 (Fig. 5 *A–C*). In the same authentic virus assay, average neutralization GMT of human convalescent plasma was 465, an approximately fivefold reduction in titer compared to the WA1/2020 strain (19). Thus, RFN vaccination elicited broadly reactive neutralizing antibody responses with potent activity against two important variants, exceeding that of convalescent individuals. Serum binding to the variant forms of SARS-CoV-2 was also assessed by biolayer interferometry (*SI Appendix*, Fig. S7). In both vaccine groups, no changes in binding to B.1.1.7 were observed, while responses to B.1.351 trended ∼15% lower.

In addition to SARS-CoV-2 VOCs, another open question in the field is the ability of existing SARS-CoV-2 vaccine platforms to protect against future SARS-CoV−like coronavirus outbreaks. Cross-protective vaccine-elicited immunity against SARS-CoV-1 may be a useful metric to address this question. We measured IgG antibody responses able to bind SARS-CoV-1 RBD by biolayer interferometry in macaque serum at week 2 following the second vaccination. All RFN-vaccinated animals developed cross-reactive binding antibodies to SARS-CoV-1 at levels approximately half those to SARS-CoV-2 (Fig. 5*D* and *SI Appendix*, Fig. S1). Binding responses were also measured to a series of SARS-CoV-1 and SARS-CoV-2 antigens, using a Luminex assay (*SI Appendix*, Fig. S8). Strong binding responses were observed to the SARS-1 S1 subunit and RBD, but not against the S2 subunit or N-terminal domain. SARS-CoV-1 RBD-specific binding antibody responses were ∼70% that of the SARS-CoV-2 response. The functional capacity of these cross-binding antibodies to mediate effector activity was assessed in an ADCP assay using SARS-CoV-1 trimeric S antigen. SARS-CoV-1 ADCP responses were observed in plasma of all vaccinated animals and were comparable between the dose groups (Fig. 5*E*).

Neutralizing titers against SARS-CoV-1 were measured using both authentic virus and pseudovirus neutralization assays, with cross-neutralizing responses observed in most RFN-vaccinated animals (Fig. 5 *F* and *G*). Substantive (GMT of 160) authentic virus neutralization titers were elicited by 50 μg of RFN 2 wk following the second immunization. SARS-CoV-1 pseudovirus neutralization activity was also observed in both the 50- and 5-μg groups, although background in a subset of control animals limited interpretation of both assays.

To assess T cell cross-reactivity to SARS-CoV-1, we evaluated whether the RFN vaccine−elicited T cells could recognize SARS-CoV-1 S. PBMCs stimulated with SARS-CoV-1 S peptide pools were stained for intracellular cytokine expression to quantitate cross-reactive T cells. Significant CD4+ T cell Th1 responses were observed following the 50-μg RFN vaccination series, though they were approximately fivefold lower in magnitude than those to SARS-CoV-2 S (Fig. 5*H*). SARS-CoV-1 S-specific CD40L responses were comparable to the Th1 responses for both dose groups (*SI Appendix*, Fig. S9*A*). IL-21 and Th2 CD4+ T cell responses were minimal or negligible (*SI Appendix*, Fig. S9 *B* and *C*). Cross-reactive CD8+ T cells were elicited and similar in magnitude to SARS-CoV-2-specific responses (∼0.1 to 0.3%) (Fig. 5*I*), suggesting that the CD8+ T cell RBD epitope specificities elicited by RFN vaccination may be conserved. Again, responses trended greater with the higher vaccine dose. These data indicate that S-specific CD4+ and CD8+ T cells generated by ALFQ-adjuvanted RFN were able to cross-react with sequence divergent SARS-CoV-1.

## Discussion

New SARS-CoV-2 vaccines may be needed to address concerns regarding emerging virus variants less sensitive to immunity elicited by current vaccines (1, 47–50). In this study, we evaluated a candidate RFN vaccine adjuvanted with ALFQ in rhesus macaques and observed robust and broad humoral and T cell responses and protection from high-dose respiratory tract challenge. Binding, neutralizing, and effector antibody responses were elicited in all animals and were of exceptional magnitude, with reciprocal peak mean neutralizing antibody titers exceeding 10^4^. S-specific CD4+ T cell responses surpassed 0.5% of memory cells and were predominantly of the Th1 phenotype. Using a rigorous challenge model in which peak viral loads of control animals achieved 10^6^ and 10^6.5^ median sgmRNA copies in the upper and lower airways, respectively, and sgmRNA persisted for 7 d, replicating virus was rapidly cleared in the airways of vaccinated animals. Absence of N-specific humoral responses following challenge in vaccinated animals further suggested little to no viral replication. Cross-reactive antibody responses were either higher or similar against the B.1.1.7 VOC in authentic and pseudovirus neutralization assays, while B.1.351 reactivity was diminished only approximately twofold. Additionally, binding and functional antibodies were also reactive to SARS-CoV-1, which is 36% amino acid sequence divergent from SARS-CoV-2 in the RBD (51). Overall, these data indicate broad, potent, and efficacious immunity elicited by RFN-ALFQ.

This study provides strong evidence that RBD-directed vaccination in primates is able to protect against SARS-CoV-2 infection and elicit neutralization breadth against variant B.1.351, which has shown the greatest resistance to neutralization by vaccinee sera (19, 52, 53). While many RBD-based immunogens have been shown to be immunogenic in small and large animal models (24–27), limited studies assessed efficacy against viral challenge and neutralization activity against VOCs. A recent macaque study investigated the immunogenicity and protective efficacy of a three-dose regimen of an RBD-ferritin nanoparticle-based vaccine (RBD scNP) and reported efficacy upon challenge, with no sgmRNA detected in the upper or lower airways of vaccinated animals at day 2 postchallenge (54). Neutralizing antibody titers against the B.1.1.7 variant were equivalent to WA1/2020, while B.1.351 reactivity was decreased threefold. Here, RFN vaccination also elicited B.1.1.7 neutralization similar to RBD scNP, and neutralized B.1.351 with twofold reduction in potency relative to WA1/2020. In another study, vaccination with RBD fused to the Fc domain of human IgG1 reduced total viral RNA following challenge in cynomolgus macaques, although virus replication was not assessed (55). On a technical note, two authentic virus neutralization assays were used in this study to measure serum activity against WA1/2020 at either 4 wk or 2 wk following the second vaccination, with VOCs included in the latter. Our intent was to compare results within rather than between assays. However, despite these differences, WA1/2020 ID_50_ values were within fivefold of one another, demonstrating consistent results in orthogonal assays. Our findings demonstrate RBD-specific immunity elicited by a two-dose vaccine regimen is protective and, importantly, cross-neutralizes the more resistant B.1.351 variant.

The immunogenicity and efficacy of ALFQ-adjuvanted RFN compares favorably to preclinical macaque data reported for three COVID-19 vaccines authorized for emergency use. RFN vaccination elicited peak mean SARS-CoV-2 pseudovirus neutralization reciprocal titers of 14,540 and 21,298 for the 5- and 50-µg groups, respectively, using an assay harmonized in a large concordance survey. Reported titers for EUA vaccines ranged from 408 to 1,862 (56–58), although results from the assays may not be directly comparable to those reported here, due to variability across laboratories. While neutralizing activity is unlikely to be the sole determinant of vaccine-mediated protection, it has been predictive of efficacy in human trials (1). Therefore, the neutralizing titers elicited by RFN relative to those elicited in NHP studies by efficacious vaccines currently in clinical use strongly suggest that RFN would be protective in humans. In addition, breadth against the B.1.351 VOC appears remarkable, as the modest approximately twofold reduction in B.1.351 neutralization activity relative to wild-type virus reported here is less than the ∼10- to 12-fold reduction in mRNA vaccinee sera as assessed by the same authentic virus neutralization assay and the same laboratory (19). The most advanced platform closest in design and composition to RFN is NVX-CoV2373, a prefusion spike nanoparticle vaccine delivered with a saponin-based Matrix-M adjuvant. NVX-CoV2373 elicited neutralizing antibody titers of 6,400 to 17,000 in macaques (59, 60). T cell immunity was also pronounced with RFN, as S-specific Th1 CD4+ T cells ranged from 0.5 to 5% following 50 µg of RFN. Reported peak values in NHPs vaccinated with EUA vaccines were 0.1 to 0.2% (56–58). Among the RFN-elicited humoral immune response measurements associated with rapid viral clearance, some novel correlates were identified, including antibodies that bind cell surface S protein, and ACE2-inhibiting antibodies, as well as ADCD, which has been reported in a previous study (16). A trend was also observed for pseudovirus neutralization, a correlate identified in several other preclinical vaccine studies (16, 58, 61–63), as well as in studies of vaccinated individuals and individuals with prior SARS-CoV-2 infection (64, 65).

The comparison of low- or high-dose vaccine regimens presented here demonstrates that immune responses did not significantly differ between the 5- and 50-µg doses, although the 50-μg group trended toward higher responses and a slightly earlier resolution of viral load. The power to detect these differences may have been limited by small sample sizes. It is likely that, as doses decrease, protective efficacy will wane, and such experiments may allow further elucidation of correlates of protection. This absence of a strong dose titration effect suggests that vaccination with the lower dose may be possible for dose-sparing purposes, although clinical testing and assessment of response durability are required. The mechanism(s) underlying the robust humoral and T cell immune responses elicited by RFN-ALFQ vaccination warrants further investigation and likely includes a combination of the ALFQ adjuvant and the highly ordered antigen array displayed on the ferritin nanoparticle, both of which stimulate adaptive immunity (22, 66). Adjuvanting ferritin nanoparticle vaccines with ALFQ increased immunogenicity relative to aluminum hydroxide in mice (66, 67), and ongoing studies in macaques aim to assess the contribution of the adjuvant to vaccine immunogenicity and efficacy in primates.

In addition to the RFN vaccine described here, we have also developed a similar ferritin nanoparticle immunogen displaying the full prefusion stabilized SARS-CoV-2 spike glycoprotein (SpFN) and reported its immunogenicity and efficacy in NHPs (68). Compared to two-dose SpFN regimens, RFN elicited binding and neutralizing antibody and T cell responses of a similar magnitude, albeit with a trend toward slightly lower titers. Postchallenge control of viral replication was also similar, although viral clearance after SpFN vaccination trended faster from the BAL at day 2 postchallenge and from NP swabs by day 4. The overall magnitude of these differences was small and suggests that both the RBD and S proteins are similarly immunogenic and protective when complexed to ferritin nanoparticles and administered with ALFQ adjuvant at these vaccine doses. A potential benefit of RBD vaccination is avoiding “antigenic sin” against nonneutralizing epitopes in the S2 subunit among antigen-experienced individuals. However, S-based immunogens may offer the advantage of broadening the specificity of the immune response to other domains and subdomains of the spike protein, limiting potential for viral escape. These findings support further clinical development of both products.

There exists a strong potential for future pandemics arising from zoonotic SARS-CoV−like *Betacoronaviruses* entering into humans. We report SARS-CoV-2 RFN vaccine−elicited responses that cross-react with the S glycoprotein of SARS-CoV-1, including binding antibody titers within an order of magnitude of those to SARS-CoV-2. The observed cross-neutralizing and binding reactivity to SARS-CoV-1 suggests that adjuvanted RFN may be a viable candidate for vaccination against future Sarbecovirus outbreaks. Work is ongoing to elucidate the potential mechanisms of cross-protective responses in this study, including epitope mapping of the antibody binding responses. Taken together, these findings support continued development of RFN vaccines for managing COVID-19 and related SARS-CoV−like virus outbreaks.

## Materials and Methods

### Vaccine and Adjuvant Production.

#### DNA plasmid construction and preparation.

The SARS-CoV-2 RBD-ferritin construct was derived from the Wuhan-Hu-1 strain genome sequence (GenBank accession number MN9089473) comprising residues 331 to 527. RBD was attached to *H. pylori* ferritin using a GSGGGG linker followed by a short sequence (ESQVRQQFSK) derived from bullfrog ferritin (69) and synthesized by GenScript, to include an N-terminal hexa-histadine (his) tag for purification. Additional point mutations (Y453R, L518N, L519K, H520S) were introduced in the RBD, using a modified QuikChange site-directed mutagenesis protocol (Agilent Technologies) and designated as construct RFN_131. The construct used a prolactin leader sequence (70). Plasmid DNA generated by site-directed mutagenesis was prepared from *Eschericia coli* Stbl3 cells. Large-scale DNA isolation was performed using either endo free Maxiprep, Megaprep, or Gigaprep kits (Qiagen).

#### Immunogen expression and purification.

SARS-CoV-2 RFN_131 immunogen (RFN) was expressed in Expi293 mammalian cell lines by transient transfection using Turbo293 transfection reagent (Speed Biosystems). Expression cultures were grown in polycarbonate baffled shaker flasks at 34 °C and 8% CO_2_ at 120 rpm. Cells were harvested 5 d posttransfection via centrifugation at 3,500 × *g* for 30 min. Culture supernatants were filtered with a 0.22-µm filter and stored at 4 °C prior to purification. RFN was purified using Ni-NTA affinity chromatography. One milliliter of Ni-NTA resin (Thermo Scientific) was used to purify protein from 1 L of expression supernatant. Ni-NTA resin was equilibrated with five column volumes (CV) of PBS (pH 7.4) followed by supernatant loading at room temperature (RT). Unbound protein was removed by washing with 200 CV of PBS, followed by 50 CV of 10 mM imidazole in PBS. Bound protein was eluted with 220 mM imidazole in PBS. Purification purity was assessed by sodium dodecyl sulfate polyacrylamide gel electrophoresis; RFN was concentrated in the presence of 5% glycerol and then further purified by size-exclusion chromatography using a 16/60 Superdex-200 purification column. Endotoxin levels for ferritin nanoparticle immunogens were evaluated (Endosafe nexgen-PTS, Charles River Laboratories), and 5% vol/vol glycerol was added prior to filter sterilization with a 0.22-µm filter, flash freezing in liquid nitrogen, and storage at −80 °C. Ferritin nanoparticle formation was confirmed by negative-stain electron microscopy and dynamic light scattering by determining the hydrodynamic diameter at 25 °C using a Malvern Zetasizer Nano S (Malvern Panalytical) equipped with a 633-nm laser.

#### Adjuvant preparation.

ALFQ formulation was prepared as previously described (71, 72). ALFQ is a unilamellar liposome comprising dimyristoyl phosphatidylcholine (DMPC), dimyristoyl phosphatidylglycerol (DMPG), cholesterol (Chol), and synthetic monophosphoryl lipid A (3D-PHAD) (Avanti Polar Lipids) and QS-21 (Desert King). DMPC and cholesterol were dissolved in chloroform, and DMPG and 3D-PHAD were dissolved in chloroform:methanol 9:1. Lipids were mixed in a molar ratio of 9:1:12.2:0.114 (DMPC:DMPG:Chol:3D-PHAD), and the solvent was removed by rotary evaporation. Lipids were suspended in Sorenson's PBS, pH 6.2, microfluidized to form small unilamellar vesicles and filtered. QS-21 was solubilized in Sorenson's PBS, pH 6.2, filtered and added to the vesicles to form ALFQ. The final lipid ratio was 9:1:12.2:0.114:0.044 (DMPC:DMPG:Chol:3D-PHAD:QS-21).

#### Immunogen formulation.

RFN was diluted in Dulbecco’s PBS to 0.1 mg/mL or 0.01 mg/mL and mixed 1:1 with 2× ALFQ on a tilted slow-speed roller at RT for 10 min, followed by incubation at 4 °C for 50 min. Reagents were equilibrated to RT before use, and immunizations were performed within 4 h of vaccine formulation. Each vaccine comprised a 1.0-mL solution of RFN formulated with ALFQ. The 3D-PHAD and QS-21 doses were 200 and 100 µg, respectively.

### Study Design and Procedures.

Twenty-three male and female specific pathogen−free, research-naïve Chinese-origin rhesus macaques (age 3 y to 7 y) were distributed—on the basis of age, weight, and sex—into three cohorts of seven or eight animals (*SI Appendix*, Table S1). Animals were vaccinated intramuscularly with either 50 μg or 5 μg of RFN, formulated with ALFQ, and control group animals received 1 mL of PBS, in the anterior proximal quadriceps muscle, on alternating sides with each dose in the series. Immunizations were administered twice, 4 wk apart. Animals were challenged 4 wk after the second immunization with virus stock obtained through BEI Resources, National Institute of Allergy and Infectious Diseases (NIAID), NIH: SARS-Related Coronavirus 2, Isolate USA-WA1/2020, NR-53780 (Lot# 70038893). Virus was stored at −80 °C prior to use, thawed by hand, and placed immediately on wet ice. Stock was diluted to 5 × 10^5^ TCID_50_/mL in PBS and vortexed gently for 5 s prior to inoculation via combined IT and IN routes (1 mL each).

All procedures were carried out in accordance with institutional, local, state, and national guidelines and laws governing animal research included in the Animal Welfare Act. Animal protocols and procedures were reviewed and approved by the Animal Care and Use Committee of both the US Army Medical Research and Development Command (USAMRDC, protocol 11355007.03) Animal Care and Use Review Office and the Institutional Animal Care and Use Committee of Bioqual, Inc. (protocol number 20-092), where NHPs were housed for the duration of the study. USAMRDC and Bioqual, Inc. are both accredited by the Association for Assessment and Accreditation of Laboratory Animal Care and are in compliance with the Animal Welfare Act and Public Health Service Policy on Humane Care and Use of Laboratory Animals. Research was conducted in compliance with the Animal Welfare Act and other federal statutes and regulations relating to animals and experiments involving animals and adheres to principles stated in *Guide for the Care and Use of Laboratory Animals* (73).

### Convalescent Plasma Samples.

A panel of 41 human convalescent-phase plasma samples was obtained from BEI Resources Repository (*n* = 30), StemExpress (*n* = 7), and a Walter Reed Army Institute of Research institutional review board-approved leukapheresis protocol (#1386H) (*n* = 4) for which written informed consent was provided by participants. Samples were collected from males (*n* = 20) and females (*n* = 21) ranging in age from 31 y to 71 y. Individuals donated plasma specimens approximately 4 to 8 wk after laboratory-confirmed SARS-CoV-2 infection and had histories of asymptomatic-to-mild-to-moderate clinical presentation. All samples were deidentified prior to use.

### Antibody Responses.

#### Binding antibodies.

SARS-CoV-2−specific binding IgG antibodies and ACE2-inhibiting antibodies were measured using MULTI-SPOT 96-well plates (MSD). Multiplex wells were coated with SARS-CoV-2 antigens, S and RBD, at a concentration of 200 to 400 ng/mL and bovine serum albumin (BSA), which served as a negative control. Four-plex MULTISPOT plates were blocked with MSD Blocker A buffer for 1 h at RT while shaking at 700 rpm. Plates were washed with buffer before the addition of reference standard and calibrator controls. Serum samples were diluted at 1:1,000 to 1:100,000 in diluent buffer, then added to each of the four wells. Plates were incubated for 2 h at RT while shaking at 700 rpm, and then washed. MSD SULFO-TAG anti-IgG antibody was added to each well. Plates were incubated for 1 h at RT with shaking at 700 rpm and washed, and then MSD GOLD Read buffer B was added to each well. Plates were read by the MESO SECTOR SQ 120 Reader. IgG concentration was calculated using DISCOVERY WORKBENCH MSD Software and reported as arbitrary units per milliliter.

For binding antibodies that block S or RBD binding to ACE2, antigen-coated plates were blocked and washed as described above. Assay calibrator and samples were diluted at 1:25 to 1:1,000 in MSD Diluent buffer, then added to the wells. Plates were incubated for 1 h at RT while shaking at 700 rpm. ACE2 protein conjugated with MSD SULFO-TAG was added, and plates were incubated for 1 h at RT while shaking at 700 rpm and washed and read as described above.

Binding antibody measurements by Octet biolayer interferometry were made using HIS1K biosensors hydrated in PBS prior to use, using an Octet FortéBio Red96 instrument (Sartorius). All assay steps were performed at 30 °C with agitation set at 1,000 rpm. Baseline equilibration of the HIS1K biosensors (Sartorius) was carried out with PBS for 15 s, prior to SARS-CoV2 RBD molecules (30 µg/mL diluted in PBS) loading for 120 s. Biosensors were dipped in assay buffer (15 s in PBS), and dipped in the serum samples (100-fold dilution) for 180 s, and binding response (nm) was recorded for 180 s.

#### Virus neutralization.

##### SARS-CoV-2 and SARS-CoV-1 pseudovirus neutralization.

The S expression plasmid sequences for SARS-CoV-2 and SARS-CoV-1 were codon optimized and modified to remove the last 18 or 28, respectively, amino acids of the cytoplasmic tail to improve S incorporation into pseudovirions (PSV). PSV were produced by cotransfection of HEK293T/17 cells with either a SARS-CoV-2 S plasmid, derived from the Wuhan-Hu-1 genome sequence (GenBank accession number MN908947.3), or a SARS-CoV-1 (Sino 1-11, GenBank accession number AY485277) S plasmid and an HIV-1 pNL4-3 luciferase reporter plasmid (pNL4-3.Luc.R-E-, NIH HIV Reagent Program, catalog number 3418). S expression plasmids for SARS-CoV-2 VOCs were similarly codon optimized and modified, and included the following mutations: B.1.1.7 (69 to 70 del, Y144del, N501Y, A570D, D614G, P681H, T718I, S982A, D1118H), B.1.351 (L18F, D80A, D215G, 241 to 243 del, K417N, E484K, N501Y, D614G, A701V, E1195Q). Infectivity and neutralization titers were determined using ACE2-expressing HEK293 target cells (Integral Molecular) in a semiautomated assay format using robotic liquid handling (Biomek NXp Beckman Coulter). Virions pseudotyped with the vesicular stomatitis virus G protein were used as a nonspecific control. Test sera were diluted 1:40 in growth medium and serially diluted; then 25 μL per well was added to a white 96-well plate. An equal volume of diluted PSV was added to each well, and plates were incubated for 1 h at 37 °C. Target cells were added to each well (40,000 cells per well), and plates were incubated for an additional 48 h. Relative light units were measured with the EnVision Multimode Plate Reader (Perkin-Elmer) using the Bright-Glo Luciferase Assay System (Promega). Neutralization dose–response curves were fitted by nonlinear regression using the LabKey Server. Final titers are reported as the reciprocal of the serum dilution necessary to achieve 50% inhibition SARS-CoV-2 (ID_50_) or 90% inhibition for SARS-CoV-1 (ID_90_, 90% inhibitory dilution). Assay equivalency was established by participation in the SARS-CoV-2 Neutralizing Assay Concordance Survey run by the Virology Quality Assurance Program and External Quality Assurance Program Oversite Laboratory at the Duke Human Vaccine Institute.

##### Authentic SARS-CoV-2 wild-type neutralization assay.

Authentic virus neutralization was measured using SARS-CoV-2 (2019-nCoV/USA_WA1/2020 [WA1/2020]) obtained from the Centers for Disease Control and Prevention and passaged once in Vero CCL81 cells (American Type Culture Collection, ATCC). Rhesus sera were serially diluted and incubated with 100 focus-forming units of SARS-CoV-2 for 1 h at 37 °C. Serum−virus mixtures were added to Vero E6 cells in 96-well plates and incubated for 1 h at 37 °C. Cells were overlaid with 1% (wt/vol) methylcellulose in Minimum Essential Medium (MEM). After 30 h, cells were fixed with 4% paraformaldehyde in PBS for 20 min at RT, and then washed and stained overnight at 4 °C with 1 µg/mL CR3022 antibody in PBS supplemented with 0.1% saponin and 0.1% BSA. Cells were subsequently stained with horseradish peroxidase-conjugated goat anti-human IgG for 2 h at RT. SARS-CoV-2−infected cell foci were visualized with TrueBlue peroxidase substrate (KPL) and quantified using ImmunoSpot microanalyzer (Cellular Technologies). Neutralization curves were generated with Prism software (GraphPad Prism 8.0).

##### Authentic SARS-CoV-2 variant and SARS-CoV-1 neutralization assay.

The SARS-CoV-2 viruses WA1/2020, USA/CA_CDC_5574/2020 (B.1.1.7), and hCoV-19/South Africa/KRISP-EC-K005321/2020 (B.1.351) were obtained from BEI Resources (NIAID, NIH) and propagated for one passage using Vero-E6 cells. Virus infectious titer was determined by an end-point dilution and cytopathic effect (CPE) assay on Vero-E6 cells to standardize the input of the different variants. In order to confirm that comparable amounts of the variants were used, the antiviral activity of Remdesivir nucleoside was tested against the different variants, and the 50% inhibition drug concentration against each virus was within the range of single dilution. In brief, serum samples were heat inactivated and subjected to successive threefold dilutions starting from 1:50. Triplicates of each dilution were incubated with SARS-CoV-2 at a multiplicity of infection of 0.1 in Eagle’s MEM with 7.5% inactivated fetal calf serum for 1 h at 37 °C. Virus–antibody mixture was transferred onto a monolayer of Vero-E6 cells grown overnight and incubated for ∼70 h. CPE of viral infection was visually scored for each well in a blinded fashion by two independent observers. Results were reported as percentage of neutralization at a given sample dilution. A SARS-CoV-1 authentic plaque reduction virus neutralization assay was performed similarly to the method previously described (74) with the following modifications. The starting dilution of serum was 1:5, and ∼100 plaque-forming units of virus were used for virus/serum incubation. The overlay used after virus adsorption was Dulbecco’s Modified Eagle Medium containing 2% fetal bovine serum (FBS) and 20% methylcellulose. Plates were then incubated for 5 d, and, post crystal violet staining, the washing step utilized water. Plaques were graded as follows: ∼25 plaques and/or 25% monolayer damage (MD) (-/+); ∼50 plaques and/or 50% MD (+); ∼75 plaques and/or 75% MD (++); ∼100 plaques and/or 100% MD (+++). All negative control wells were solid monolayers.

#### ADNP.

Biotinylated SARS-CoV-2 prefusion stabilized S trimer was incubated with yellow-green streptavidin-fluorescent beads (Molecular Probes) for 2 h at 37 °C. Ten microliters of a 100-fold dilution of protein-coated beads was incubated for 2 h at 37 °C with 100 μL of 8,100-fold diluted plasma samples before addition of effector cells (50,000 cells per well). Fresh human PBMCs were used as effector cells after red blood cell lysis with Ammonium-Chloride-Potassium (ACK) lysing buffer (ThermoFisher Scientific). After 1 h incubation at 37 °C, cells were washed, surface stained, and fixed with 4% formaldehyde solution (Tousimis), and fluorescence was evaluated on an LSRII flow cytometer (BD Bioscience). Antibodies used for flow cytometry included anti-human CD3 AF700 (clone UCHT1), anti-human CD14 APC-Cy7 (clone MϕP9) (BD Bioscience, San Jose, C), and anti-human CD66b Pacific Blue (clone G10F5) (BioLegend). The phagocytic score was calculated by multiplying the percentage of bead-positive neutrophils (SSC high, CD3− CD14− CD66+) by the geometric mean of the fluorescence intensity of bead-positive cells and dividing by 10,000.

#### ADCP.

ADCP was measured as previously described (75). Briefly, biotinylated SARS-CoV-1 or SARS-CoV-2 prefusion-stabilized S trimer was incubated with red streptavidin-fluorescent beads (Molecular Probes) for 2 h at 37 °C. Ten microliters of a 100-fold dilution of beads–protein was incubated for 2 h at 37 °C with 100 μL of 8,100-fold (SARS-CoV-2) or 900-fold (SARS-CoV-1) diluted plasma samples before addition of THP-1 cells (20,000 cells per well; Millipore Sigma). After 19 h incubation at 37 °C, the cells were fixed with 2% formaldehyde solution (Tousimis), and fluorescence was evaluated on an LSRII flow cytometer (BD Bioscience). The phagocytic score was calculated by multiplying the percentage of bead-positive cells by the geometric mean of the fluorescence intensity of bead-positive cells and dividing by 10,000.

#### Opsonization.

SARS-CoV-2 S-expressing expi293F cells were generated by transfection with linearized plasmid (pcDNA3.1) encoding codon-optimized full-length SARS-CoV-2 S protein matching the amino acid sequence of the IL-CDC-IL1/2020 isolate (GenBank accession number MN988713). Stable transfectants were single-cell sorted and selected to obtain a high-level S surface expressing clone (293F-spike-S2A). SARS-CoV-2 S-expressing cells were incubated with 200-fold diluted plasma samples for 30 min at 37 °C. Cells were washed twice and stained with anti-human IgG PE, anti-human IgM Alexa Fluor 647, and anti-human IgA FITC (Southern Biotech). Cells were then fixed with 4% formaldehyde solution, and fluorescence was evaluated on an LSRII flow cytometer (BD Bioscience).

#### ADCD.

ADCD was adapted from methods described previously (76). Briefly, SARS-CoV-2 S-expressing expi293F cells were incubated with 10-fold diluted, heat-inactivated (56 °C for 30 min) plasma samples for 30 min at 37 °C. Cells were washed twice and resuspended in Roswell Park Memorial Institute (RPMI) 1640 medium containing 10% FBS (R10 media). During this time, lyophilized guinea pig complement (CL4051, Cedarlane) was reconstituted in 1 mL of cold water and centrifuged for 5 min at 4 °C to remove aggregates. Cells were washed with PBS and resuspended in 200 μL of guinea pig complement, which was prepared at a 1:50 dilution in Gelatin Veronal Buffer with Ca^2+^ and Mg^2+^ (IBB-300X, Boston BioProducts). After incubation at 37 °C for 20 min, cells were washed in PBS 15 mM (ethylenedinitrilo)tetraacetic acid (ThermoFisher Scientific) and stained with an anti-Guinea Pig Complement C3 FITC (polyclonal, ThermoFisher Scientific). Cells were then fixed with 4% formaldehyde solution, and fluorescence was evaluated on an LSRII flow cytometer (BD Bioscience).

#### Trogocytosis.

Trogocytosis was measured using a previously described assay (40). Briefly, SARS-CoV-2 S-expressing expi293F cells were stained with PKH26 (Sigma-Aldrich). Cells were then washed with and resuspended in R10 media. Cells were then incubated with 200-fold diluted plasma samples for 30 min at 37 °C. Effector PBMCs were next added to the R10 media at an effector to target cell ratio of 50:1 and then incubated for 5 h at 37 °C. After the incubation, cells were washed, stained with live/dead aqua fixable cell stain (Life Technologies), and CD14 APC-Cy7 (clone MϕP9) for 15 min at RT, washed again, and fixed with 4% formaldehyde (Tousimis) for 15 min at RT. Fluorescence was evaluated on an LSRII flow cytometer (BD Bioscience). Trogocytosis was evaluated by measuring the PKH26 mean fluorescence intensity of the live CD14+ cells.

### Antigen-Specific T Cell Responses.

Cryopreserved PBMCs were thawed and rested for 6 h in R10 with 50 U/mL Benzonase Nuclease (Sigma-Aldrich). They were then stimulated with peptide pools for 12 h. Stimulations consisted of two pools of peptides spanning the S protein of SARS-CoV-2 or SARS-CoV-1 (1 µg/mL, JPT Peptide Technologies, PM-WCPV-S and PM-CVHSA-S, respectively) in the presence of Brefeldin A (0.65 µL/mL, GolgiPlug, BD Cytofix/Cytoperm Kit, catalog number 555028), costimulatory antibodies anti-CD28 (BD Biosciences catalog number 555725; 1 µg/mL) and anti-CD49d (BD Biosciences catalog number 555501; 1 μg/mL), and CD107a (H4A3, BD Biosciences catalog number 561348, lots 9143920 and 253441). Following stimulation, cells were stained serially with LIVE/DEAD Fixable Blue Dead Cell Stain (ThermoFisher #L23105) and a mixture of fluorescent-labeled antibodies (BD Biosciences unless otherwise indicated) to cell surface markers CD4-PE-Cy5.5 (S3.5, ThermoFisher #MHCD0418, lots 2118390 and 2247858), CD8-BV570 (RPA-T8, BioLegend #301038, lot B281322), CD45RA BUV395 (5H9, #552888, lots 154382 and 259854), CD28 BUV737 (CD28.2, #612815, lot 0113886), CCR7-BV650 (GO43H7, #353234, lots B297645 and B316676), and HLA-DR-BV480 (G46-6, #566113, lot 0055314). Intracellular cytokine staining was performed following fixation and permeabilization (BD Cytofix/Cytoperm, BD Biosciences) with CD3-Cy7APC (SP34-2, #557757, lots 6140803 and 121752), CD154-Cy7PE (24-31, BioLegend #310842, lots B264810 and B313191), IFNγ-AF700 (B27, #506516, lots B187646 and B290145), TNFα-FITC (MAb11, #554512, lot 15360), IL-2-BV750 (MQ1-17H12, BioLegend #566361, lot 0042313), IL-4 BB700 (MP4-25D2, lots 0133487 and 0308726), MIP-1b-PE (D21-1351, #550078, lot 9298609), CD69-ECD (TP1.55.3, Beckman Coulter #6607110, lots 7620070 and 7620076), IL-21-AF647 (3A3-N2.1, #560493, lots 9199272 and 225901), IL-13-BV421 (JES10-5A2, #563580, lots 9322765, 210147, and 169570), and IL-17a-BV605 (BL168, BioLegend #512326, lot B289357). Sample staining was measured on a FACSymphony A5 SORP (Becton Dickenson), and data were analyzed using FlowJo v.9.9 software (Tree Star, Inc.). CD4+ and CD8+ T cell subsets were pregated on memory markers prior to assessing cytokine expression as follows: single positive or double negative for CD45RA and CD28. Boolean combinations of cells expressing one or more cytokines were used to assess the total S-specific response of memory CD4+ or CD8+ T cells. Responses from the two-peptide pools spanning SARS-CoV-2 S or SARS-CoV-1 S were summed. Display of multicomponent distributions was performed with SPICE v6.0 (NIH).

### Total and sgmRNA Quantification.

Quantitative reverse transcription PCR (RT-qPCR) was carried out for sgmRNA and total viral load RNA quantification from NP swab, BAL fluid, and saliva samples. Primers targeted the envelope (E) gene of SARS-CoV-2 (*SI Appendix*, Table S2). RNA was extracted from 200 μL of specimen using the EZ1 DSP Virus kit (Qiagen) on the EZ1 Advanced XL instrument (Qiagen). Briefly, samples were lysed in 200 μL of ATL buffer (Qiagen) and transferred to the Qiagen EZ1 for extraction. Bacteriophage MS2 (ATCC) was added to the RNA carrier and used as an extraction control to monitor efficiency of RNA extraction and amplification (77). Purified RNA was eluted in 90 μL of elution buffer (AVE). The RT-qPCR amplification reactions were performed in separate wells of a 96-well Fast plate for the three targets: sgmRNA, RNA viral load, and MS2 RNA using 10 μL of extracted material 0.72 μM of primers, 0.2 μM of probe and 1× TaqPath 1-Step RT-qPCR (Life Technologies, Thermo Fisher Scientific, Inc.). Amplification cycling conditions were 2 min at 25 °C, 15 min at 50 °C, 2 min at 95 °C, and 45 cycles of 3 s at 94 °C and 30 s at 55 °C with fluorescence read at 55 °C. An RNA transcript for the SARS-CoV-2 E gene was used as a calibration standard. RNA copy values were extrapolated from the standard curve and multiplied by 45 to obtain RNA copies per milliliter. A negative control (PBS) and two positive controls, contrived using heat-inactivated SARS-CoV-2 (ATCC, VR-1986HK), at 10^6^ and 10^3^ copies per mL, were extracted and used to assess performance of both assays.

### Histopathology.

Formalin-fixed sections of lung tissue were evaluated by light microscopy and IHC. Lungs were perfused with 10% neutral-buffered formalin. Lung sections were processed routinely into paraffin wax, and then sectioned at 5 µm, and resulting slides were stained with hematoxylin and eosin. IHC was performed using the Dako Envision system (Dako Agilent Pathology Solutions). Briefly, after deparaffinization, peroxidase blocking, and antigen retrieval, sections were covered with a mouse monoclonal anti-SARS-CoV nucleocapsid protein (#40143-MM05, Sino Biological) at a dilution of 1:4,000 and incubated at RT for 45 min. They were rinsed, and the peroxidase-labeled polymer (secondary antibody) was applied for 30 min. Slides were rinsed, and a brown chromogenic substrate 3,3′ diaminobenzidine solution (Dako Agilent Pathology Solutions) was applied for 8 min. The substrate–chromogen solution was rinsed off the slides, and slides were counterstained with hematoxylin and rinsed. The sections were dehydrated, cleared with Xyless, and then coverslipped. Tissue section slides were evaluated by a board-certified veterinary anatomic pathologist who was blinded to study group allocations. IHC was performed with Dako Envision. Three tissue sections from each of the right and left lung lobes were used to evaluate the lung pathology. The histopathology of each section was evaluated on a scale of 0 to 5: 0, absent; 1, minimal (<10% of tissue section affected); 2, mild (11 to 25% of tissue section affected); 3, moderate (26 to 50% of tissue section affected); 4, marked (51 to 75% affected); 5, severe (>75% of tissue section affected). Sections were evaluated for edema, hyaline membranes, cellular infiltrates, alveolar histiocytes, type II pneumocyte hyperplasia, interstitial fibroplasia, bronchus-associated lymphoid tissue hyperplasia, bronchiolar degeneration, megakaryocytes in capillaries, and thrombosis. The scores for all six sections of each pathologic finding were combined to create the final score (Type II pneumocyte hyperplasia [TIIPH] score) for individual animals.

### Statistical Analysis.

Primary immunogenicity outputs of binding and neutralizing antibody titers as well as T cell responses were compared across vaccination groups using the Kruskal−Wallis test. Nonparametric pair-wise comparisons between groups were made using the post hoc Dunn’s test. Statistical significance was preset at an alpha level of 0.05.

## Supplementary Material

Supplementary File

## Data Availability

All study data are included in the article and *SI Appendix*. Some study data are available upon request to K.M. or D.L.B.
