# Supplementary material for: Efficacy and breadth of adjuvanted SARS-CoV-2 receptor-binding domain nanoparticle vaccine in macaques
Source: Proc Natl Acad Sci U S A. 2021 Sep 1;118(38):e2106433118. doi: 10.1073/pnas.2106433118 (PMC8463842; doi:10.1073/pnas.2106433118)
Supplement: Supplementary File [file pnas.2106433118.sapp.pdf]

Supplemental Table 1. Nonhuman primate age, sex and weight distribution by group

ID	Sex	Age in Years	Weight (kg)	Group*	Immunogen
HS1608116	F	4.03	4.54	1-A	PBS
171230	F	6.4	6.68	1-A	PBS
HS1605516	F	4.24	4.5	1-A	PBS
180231	M	4.37	7.28	1-A	PBS
HS1610040	F	3.88	3.95	1-B	PBS
HS1606329	F	4.17	4.14	1-B	PBS
HS1606025	M	4.23	3.95	1-B	PBS
HS1606397	M	4.16	3.84	1-B	PBS
171276	F	6.48	6.66	4-A	50 μg RFN
HS1606381	M	4.16	4.22	4-A	50 μg RFN
HS1606379	M	4.16	4.25	4-A	50 μg RFN
HS1704246	F	3.35	4.16	4-B	50 μg RFN
HS1607114	F	4.13	3.65	4-B	50 μg RFN
HS1603140	F	4.46	3.4	4-B	50 μg RFN
HS1603067	M	4.47	3.8	4-B	50 μg RFN
HS1606371	M	4.16	3.94	4-B	50 μg RFN
HS1610052	F	3.88	4.5	5-A	5 μg RFN
180654	F	5.46	4.16	5-A	5 μg RFN
HS1606341	M	4.17	4.25	5-A	5 μg RFN
180288	M	4.46	8.44	5-A	5 μg RFN
HS1704226	F	3.35	3.7	5-B	5 μg RFN
HS1610030	F	3.88	4.1	5-B	5 μg RFN
HS1606389	M	4.16	3.94	5-B	5 μg RFN

*A and B cohorts were sacrificed at day 14 and day 7 post-challenge, respectively

Supplemental Table 2. Primers and probes for SARS-CoV-2 sgmRNA and total RNA viral load

Primer/Probe Name	Sequence 5'- 3'	Nucleotide Length
SARS-CoV-2 TAL E1 F	TCGTGGTATTCTTGCTAG	18
SARS-CoV-2 TAL E1 R	GAAGGTTTTACAAGACTCAC	20
SARS-CoV-2 TALE1 Probe	FAM -ACACTAGCCATCCTTACTGCG-BHQ1	21
SARS-CoV-2 sg Leader	CGATCTCTTGTAGATCTGTTCTC	23
MS2 F	CTCTGAGAGCGGCTCTATTGG	21
MS2 R	GTTCCCTACAACGAGCCTAAATTC	24
MS2 Probe	JOE-TCAGACACGCGGTCCGCTATAACGAT- BHQ2	26

F = Forward Primer, R = Reverse Primer

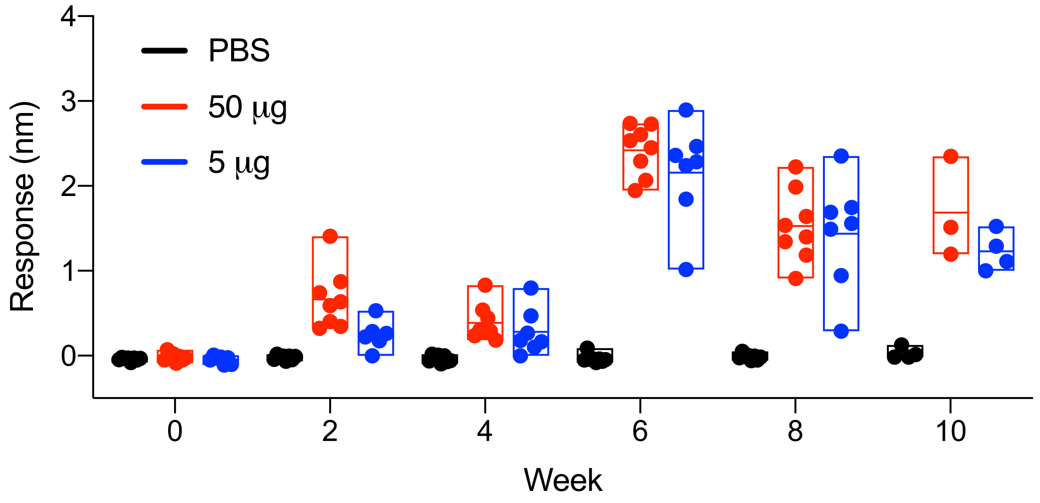

Supplemental Figure 1. Binding antibody responses to SARS-CoV-2 RBD measured by biolayer interferometry. SARS-CoV-2 RBD-specific binding antibody responses were assessed in macaque serum every two weeks following RFN immunization (weeks 0, 4) and challenge (week 8).

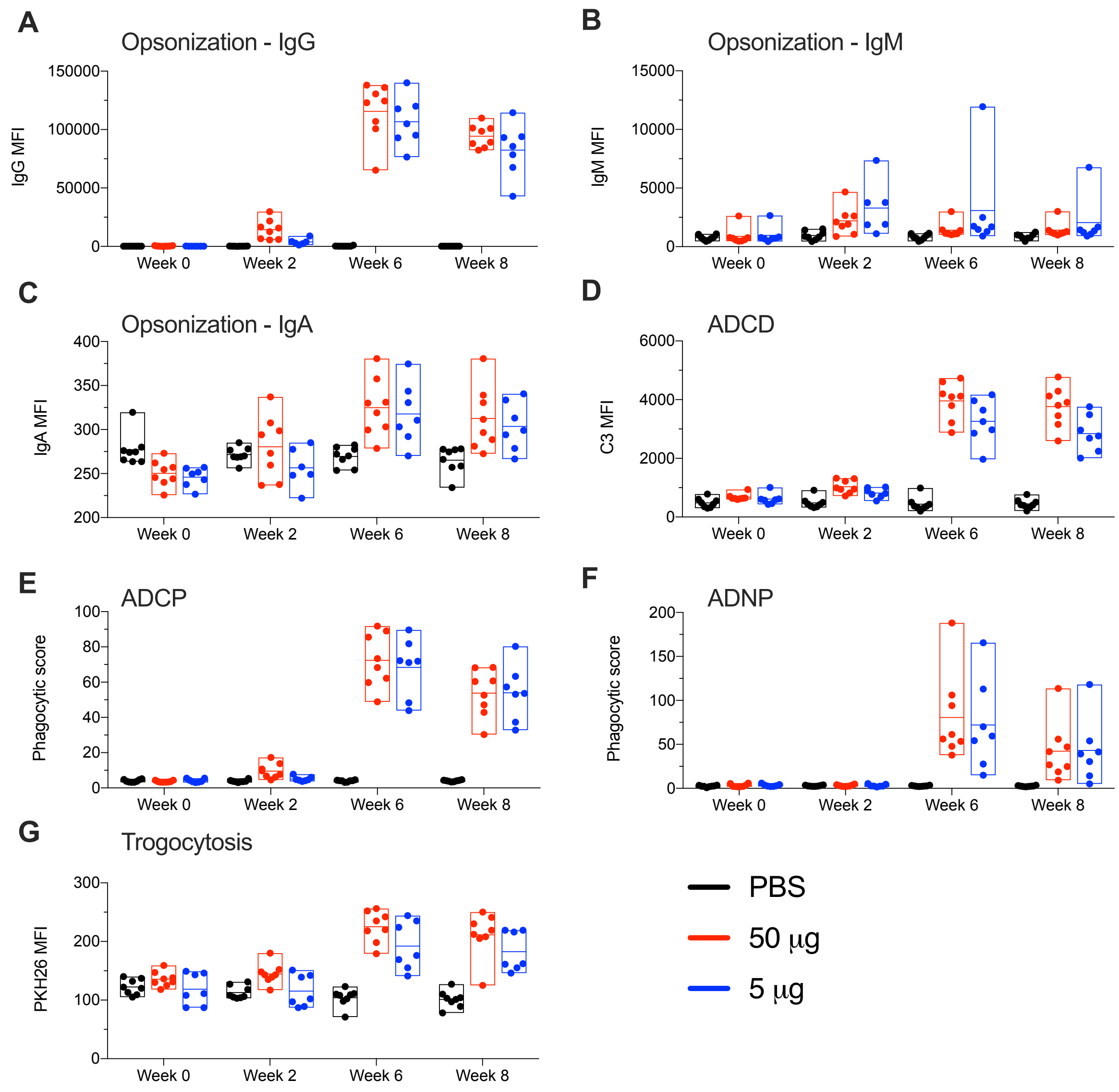

Supplemental Figure 2. Fc-mediated effector antibody responses induced by vaccination with RFN. SARS-CoV-2 (USA-WA1) S-specific plasma antibody effector activity was measured in RFN vaccinated macaques at the indicated study weeks. **(***A-C***)** Antibody-mediated cellular opsonization activity was measured using S-expressing cells incubated with diluted plasma followed by IgG (*A*), IgM (*B*), and IgA (*C*) staining detected by flow cytometry. **(***D***)** ADCD was measured on plasma-opsonized S-expressing cells. (*E,F*) ADCP and ADNP responses assessed by incubating spike-trimer-coated fluorescent beads with diluted plasma and culture with effector cells. (*G*) Trogocytosis was measured using plasma-opsonized S-expressing cells.

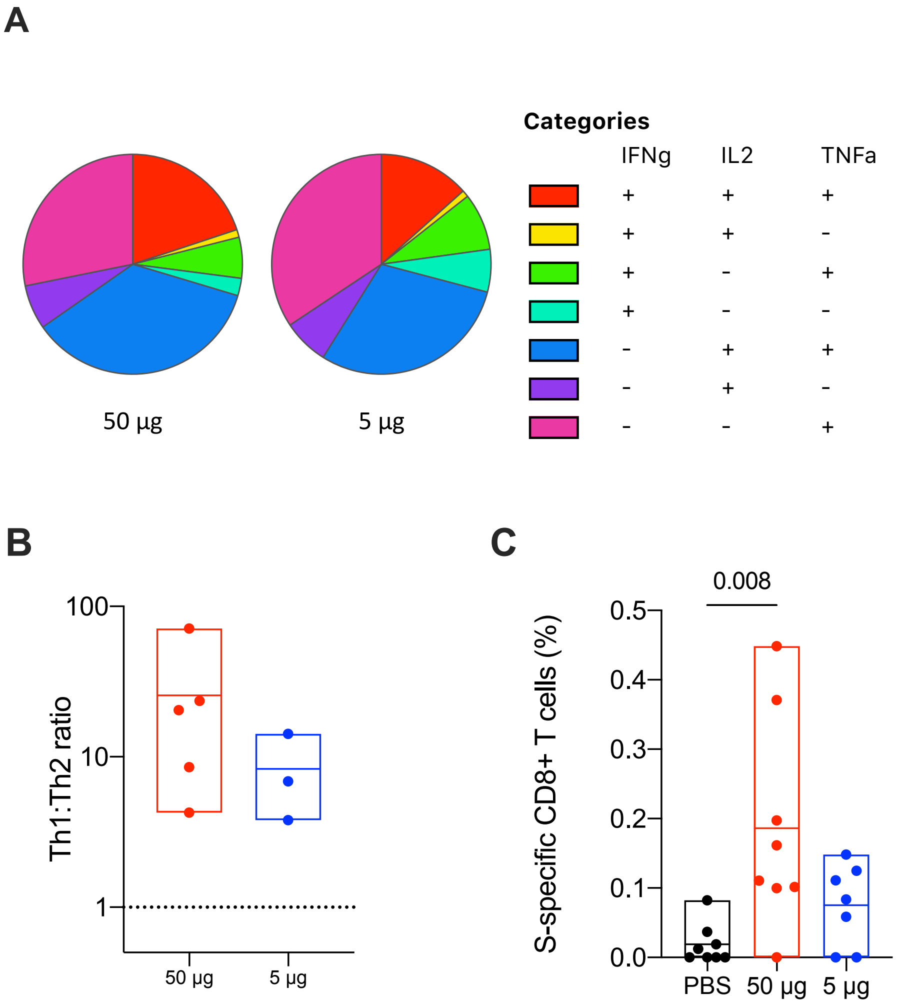

Supplemental Figure 3. CD8+ T cell responses and CD4+ T helper response ratio and polyfunctionality. SARS-CoV-2 (USA-WA1) S-specific T cell responses were assessed in PBMC of RFN vaccinated rhesus macaques four weeks after the last immunization by intracellular cytokine staining (*A*) S-specific CD4+ T cell Th1 cytokine polyfunctionality was assessed by Boolean combination gating of IFNγ, IL-2, and TNF expression. (*B*) The ratio of Th1 to Th2 S-specific memory CD4+ T cells in animals with positive Th2 responses. Dashed line indicates an equal proportion of Th1 and Th2 cells. (*C*) Memory CD8+ T cell responses were measured by stimulation with overlapping SARS-CoV-2 S peptides and IFNγ, IL-2 and TNF intracellular staining. Significance was assessed using a Kruskal-Wallis test followed by a Dunn's post-test.

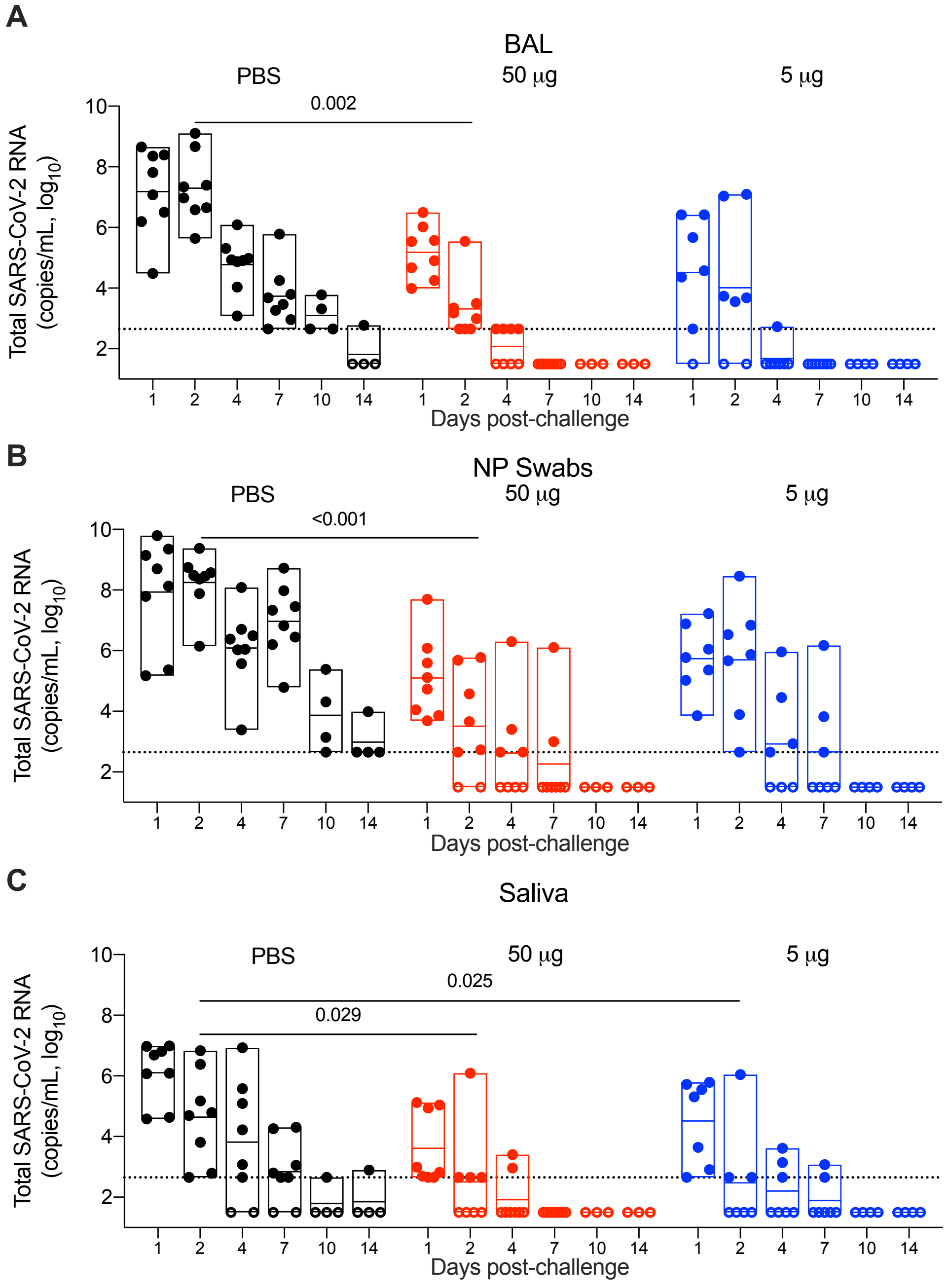

Supplemental Figure 4. Total viral load in the airways following SARS-CoV-2 respiratory tract challenge. Total SARS-CoV-2 RNA for the E (Envelope) target (copies per milliliter) were measured in bronchoalveolar lavage fluid (*A*), nasopharyngeal swabs (*B*) and saliva (*C*) of vaccinated and control animals for two weeks following intranasal and intratracheal SARS-CoV-2 (USA-WA1/2020) challenge. Specimens were collected 1, 2, 4, 7, 10 and 14 days post-challenge (N=7-8 per group for days 1-7; N=3-4 days 10 and 14). Dotted lines demarcate assay lower limit of linear performance range (log10 of 2.65 corresponding to 450 copies/mL); positive values below this limit are plotted as 450 copies/ml. Open symbols represent animals with viral loads below the limit of detection of the assay. Box plot horizontal lines indicate the mean; top and bottom reflect the minimum and maximum. Significant differences between control and vaccinated animals at day 2 post-challenge are indicated. Significance was assessed using a Kruskal-Wallis test followed by a Dunn's post-test.

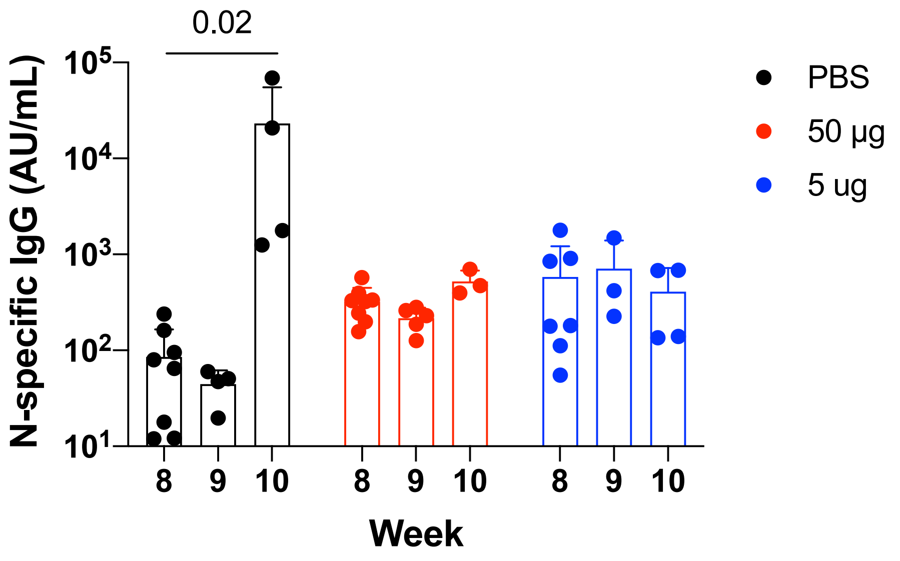

Supplemental Figure 5. N-specific IgG responses following SARS-CoV-2 challenge. Serum IgG responses to SARS-CoV-2 N assessed by MSD immunoassay at the time of challenge (week 8) and at the time of necropsy (either weeks 9 or 10). Data are depicted as the arbitrary units (AU)/ml of IgG binding. Significance was assessed using a Kruskal-Wallis test followed by a Dunn's post-test, comparing weeks 9 or 10 to baseline week 8 measurements.

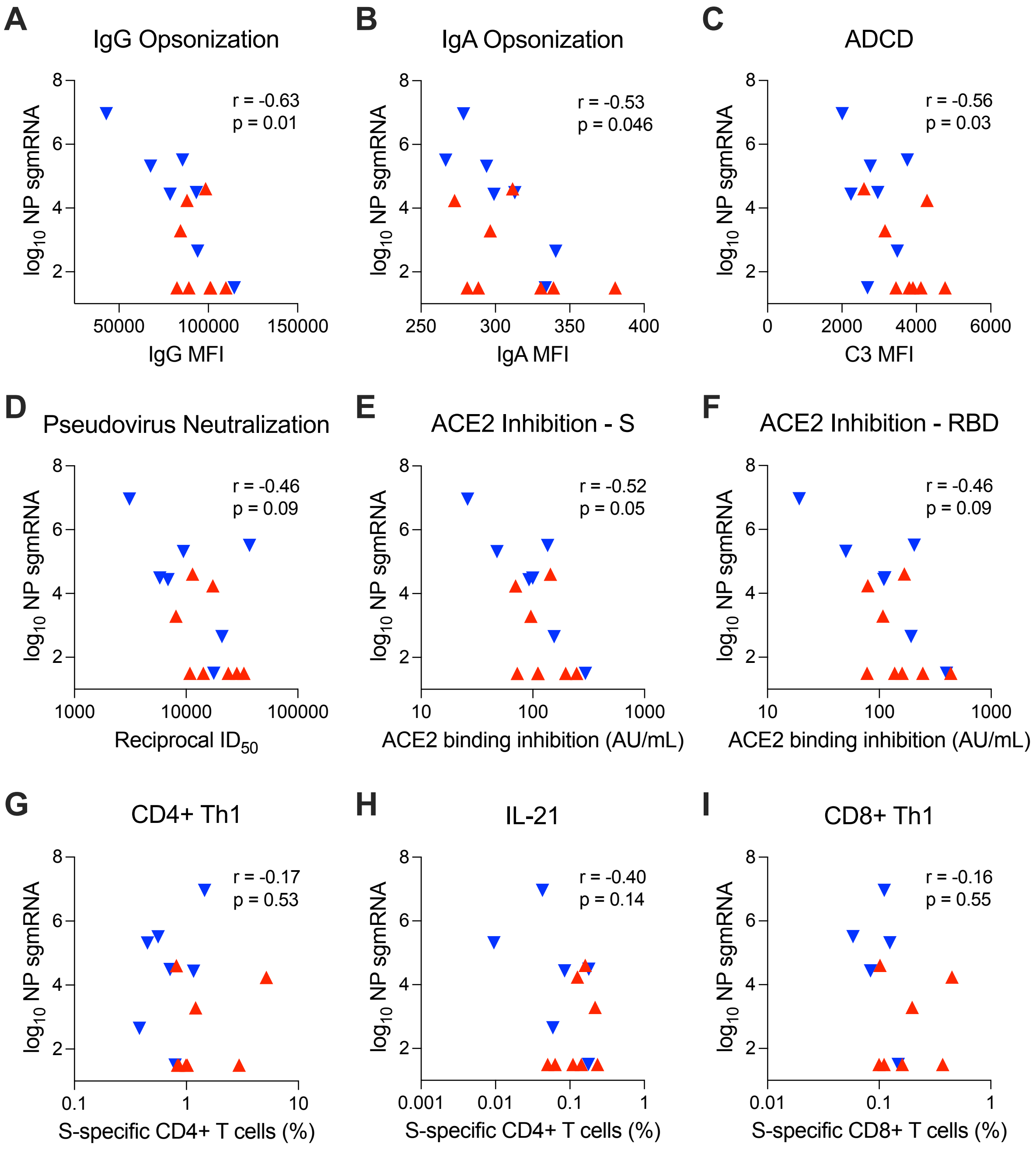

Supplemental Figure 6. Association of vaccine-elicited immune responses with viral control. Correlation between decreased viral replication in the NP swabs at day 2 post-challenge and SARS-CoV-2-specific immune responses at the time of challenge (week 8). Bivariate plots are shown for (*A*) IgG opsonization, (*B*) IgA opsonization, (*C*) ADCD, (*D*) pseudovirus neutralization, (*E*) inhibition of ACE2 binding to S, (*F*) inhibition of ACE2 binding to RBD, (*G*) S-specific CD4+ Th1 cells, (*H*) S-specific CD4+ IL-21+ cells and (*I*) S-specific CD8+ Th1 cells. Spearman's rank-order correlation (two-tailed test) rho and p values are shown. Red and blue symbols indicate animals vaccinated with 50 µg and 5 µg RFN, respectively.

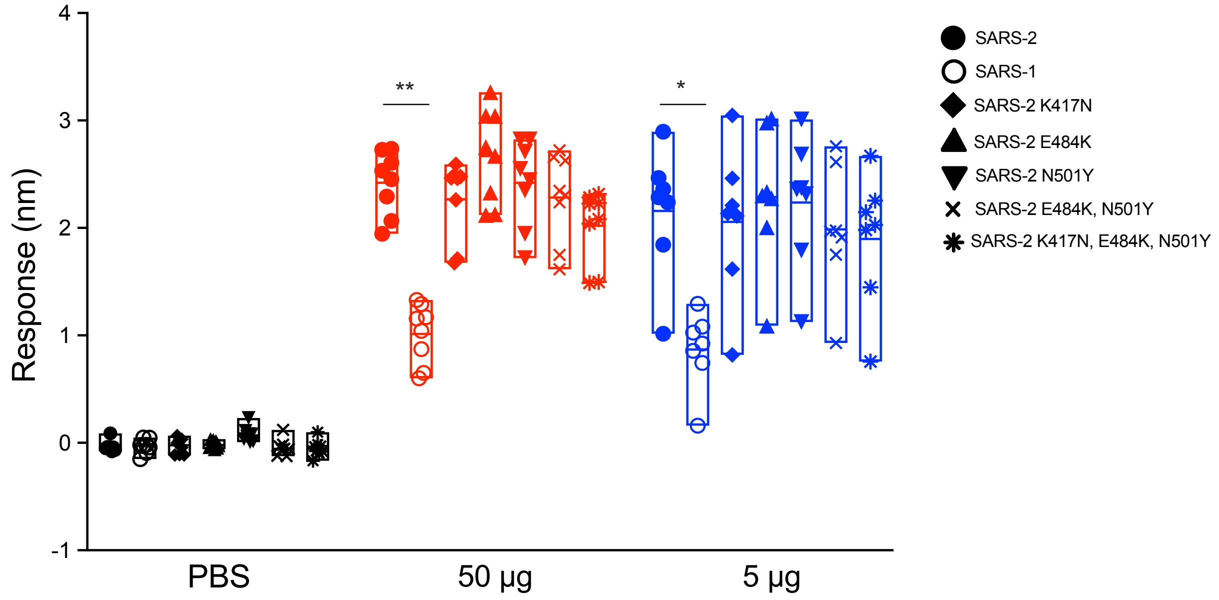

Supplemental Figure 7. Binding antibody responses to SARS-CoV-1, SARS-CoV-2 wild-type and SARS-CoV-2 mutant RBD. Serum RBD-specific antibody responses were assessed by biolayer interferometry two weeks after last RFN vaccination**.** SARS-CoV-2 RBD variant forms produced by site-directed mutagenesis were used as antigens. Significant differences relative to SARS CoV-2 wild-type binding assessed using a Kruskal-Wallis test followed by a Dunn's post-test is indicated (* <0.05; ** < 0.01).

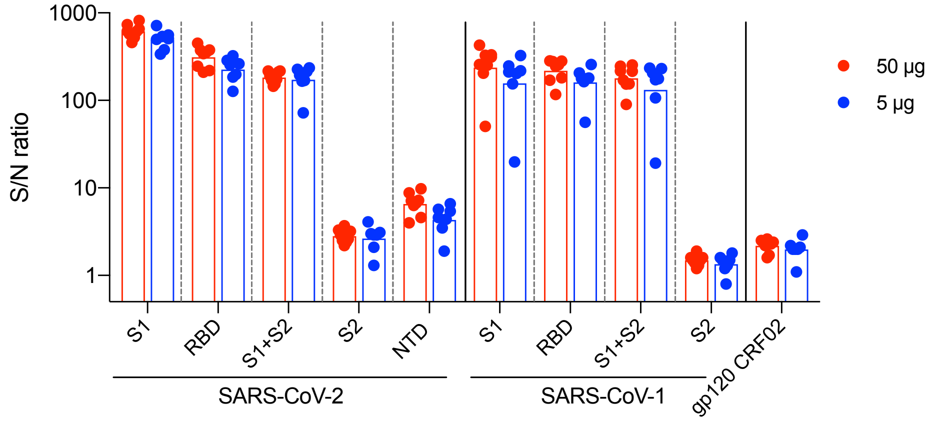

Supplemental Figure 8. Binding antibody responses to SARS-CoV-2 and SARS-CoV-1 antigens measured by Luminex. RFN vaccinated macaque plasma collected two weeks after the last immunization was evaluated for binding to SARS-CoV-2 and SARS-CoV-1 S1 and S2 subunits, RBD, and N-terminal domain (NTD) using a multiplex Luminex assay. Mean fluorescence intensity (MFI) data were divided by pre-immunization background MFI to obtain a signal to noise (S/N) ratio of vaccine-elicited binding antibody response magnitude for each sample. Antibody binding to HIV gp120 circulating recombinant form (CRF) 02 was used as a negative control antigen. Bars indicate the geometric mean.

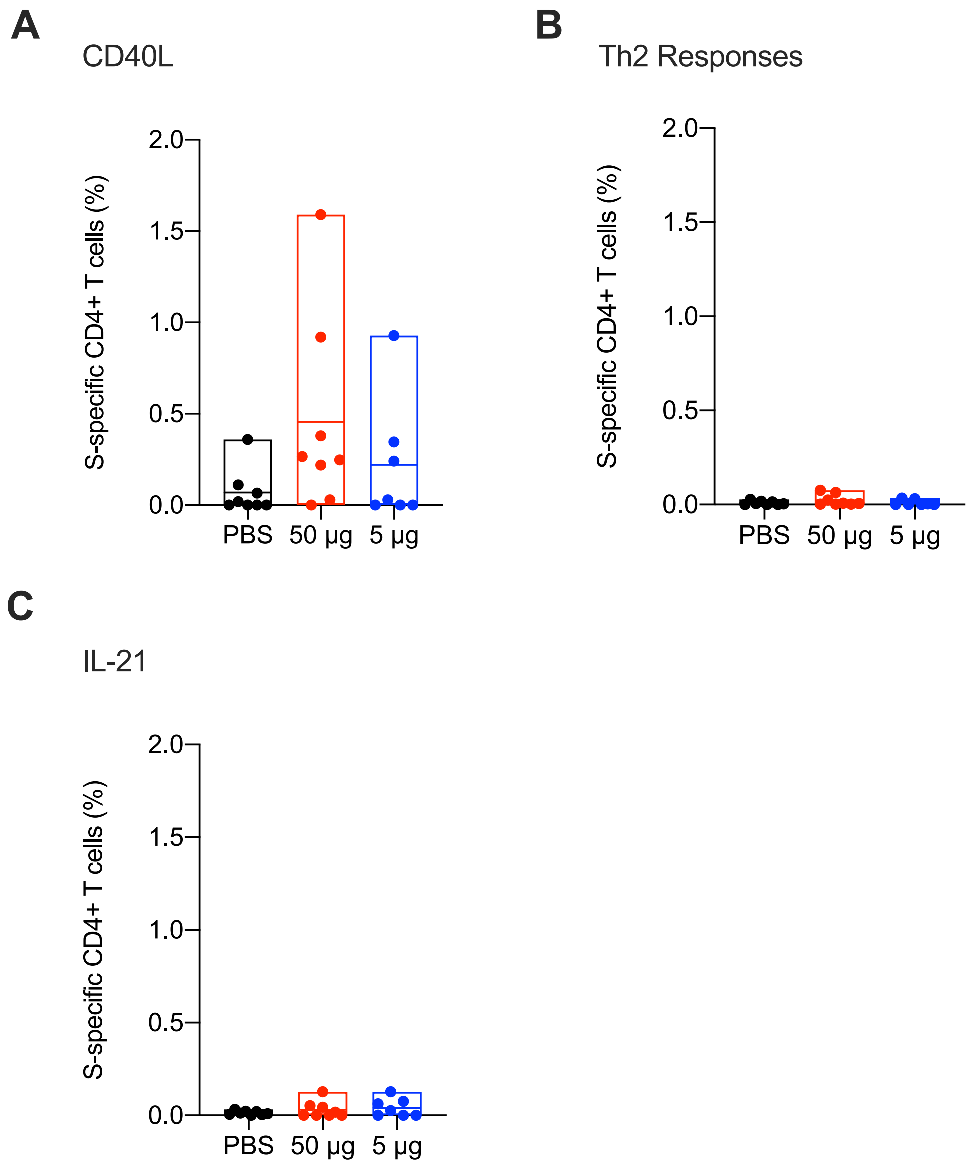

Supplemental Figure 9. Cross-reactive CD4+ T cell responses against SARS-CoV-1. Antigen-specific T cell responses were assessed in RFN vaccinated and control macaques by SARS-CoV-1 S peptide pool stimulation of PBMC collected two weeks after the last vaccination followed by ICS. The frequency of S-specific memory CD4+ T cells expressing the indicated marker(s) is shown for (*A*) CD40L, (*B*) Th2 cytokines (IL-4 and IL-13) and (*C*) IL-21. Boolean combinations of cytokine positive memory CD4+ T cells were summed. Significance was assessed using a Kruskal-Wallis test followed by a Dunn's post-test.
